# An insight into new glycotherapeutics in glial inflammation: Understanding the role of glycosylation in mitochondrial function and acute to the chronic phases of inflammation

**DOI:** 10.1111/cns.14016

**Published:** 2022-11-15

**Authors:** Vaibhav Patil, Raghvendra Bohara, Carla Winter, Michelle Kilcoyne, Siobhan McMahon, Abhay Pandit

**Affiliations:** ^1^ CÚRAM, SFI Research Centre for Medical Devices University of Galway Galway Ireland; ^2^ Microbiology University of Galway Galway Ireland; ^3^ Anatomy Galway Ireland

## Abstract

**Introduction:**

Glycosylation plays a critical role during inflammation and glial scar formation upon spinal cord injury (SCI) disease progression. Astrocytes and microglia are involved in this cascade to modulate the inflammation and tissue remodeling from acute to chronic phases. Therefore, understating the glycan changes in these glial cells is paramount.

**Method and results:**

A lectin microarray was undertaken using a cytokine‐driven inflammatory mixed glial culture model, revealing considerable differential glycosylation from the acute to the chronic phase in a cytokine‐combination generated inflamed MGC model. It was found that several N‐ and O‐linked glycans associated with glia during SCI were differentially regulated. Pearson's correlation hierarchical clustering showed that groups were separated into several clusters, illustrating the heterogenicity among the control, cytokine combination, and LPS treated groups and the day on which treatment was given. Control and LPS treatments were observed to be in dense clusters. This was further confirmed with lectin immunostaining in which GalNAc, GlcNAc, mannose, fucose and sialic acid‐binding residues were detected in astrocytes and microglia. However, the sialyltransferase inhibitor inhibited this modification (upregulation of the sialic acid expression), which indeed modulates the mitochondrial functions.

**Conclusions:**

The present study is the first functional investigation of glycosylation modulation in a mixed glial culture model, which elucidates the role of the glycome in neuroinflammation in progression and identified potential therapeutic targets for future glyco therapeutics in neuroinflammation.

## Graphical abstract

Workflow describing steps involved in the lectin microarray to understand the glycosignature in the mixed culture model.

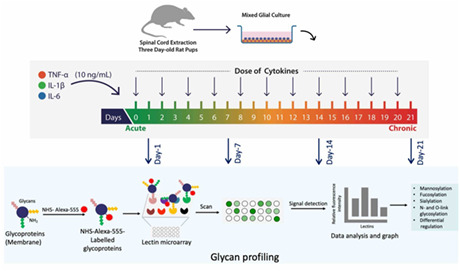

## 1 INTRODUCTION

Central nervous system (CNS) trauma such as spinal cord injury (SCI) is dominated by inflammation with the predominance of cytokines such as TNF‐alpha, IL‐1beta and IL‐6.^1^, ^2^ These cytokines further activate astrocytes and microglia to modulate their functions based on the expression of various glycans either on the surface or inside the cell. As the injury progresses, glial cells' activation and characteristics change from acute to chronic phases. Most in vitro studies deals with only the acute phase (24 h) of glial cells.^3^ However, we previously reported a mixed glial in vitro model to study acute and chronic inflammatory phases associated with SCI.^4^ Another area studied is glial scar formation after SCI and its effect on the injury's progression. The glial scar mainly comprises cells such as scar‐forming reactive astrocytes, foam cells, microglia, M1 macrophages, etc. and extracellular matrix (ECM).^5^, ^6^ Among these cells, the astrocytes are mainly responsible for the glial scar formation and secretion of ECM components.^7^ The glial scar comprises of proteoglycans and glycoproteins, further impedes neuronal regeneration.^5^, ^8^ The glial scar's major constituents include chondroitin sulphate (CS) and keratan sulphate (KS) proteoglycans. These proteoglycans are made up of glycans which are polysaccharides with distinct carbohydrate chains.^9^, ^10^ Glycans are mainly composed of N‐acetylgalactosamine (GalNAc), N‐acetylglucosamine (GlcNAc), fucose, mannose, sialic acid, galactose, lactose, or other monosaccharides.^11^ Among these, hypersialylation is a characteristic feature in which sialyltransferase increases (polysialyltransferases) and catalyzes different sialic acid linkages such as α‐(2–6, or, 2–8) on glycan chains.^12^ In vertebrates, glycans usually end with a sialic acid (sialylation) on alpha‐3 or 6 position linkage^13^ which supports molecular identification.

The modification of proteins by incorporating glycans that occurs during post‐translation transformation in the cytoplasm and endoplasmic reticulum is called glycosylation.^14^ In cellular processes, glycans play a significant role in defining the functionality of the protein or lipid. Post‐translation modification of proteins and lipids is critical in pathological conditions, and aberrant changes in these modifications could alter their functionality.^15^, ^16^, ^17^ In addition, as stated earlier, in inflammatory conditions, the activated astrocytes and microglia modulate their functions based on the expression of various glycans either on the surface or inside the cell.^17^, ^18^ It has been noted that GlcNAc and GalNAc moieties increased after SCI and mainly were found to be associated with microglia^19^, ^20^ and astrocytes.^21^ Upon microglial stimulation, they exhibit pro‐ and anti‐inflammatory responses, which are well regulated by the glycans.^22^ These glycans interact with their ligands known as lectins. Therefore, lectins are used to study glycan expression and identify specific glycan modifications either on the cell surface or intracellularly and on proteins or lipids. Lectins are the carbohydrate‐binding moieties that recognize cell surface glycan structures, and they are involved in cell‐to‐cell communication via lectin‐glycan interactions.^23^ This biomarker approach was used to study the progression of inflammatory phases from acute to chronic in the MGC model.

As mentioned in Ref. [4], using an in vitro mixed glial culture (MGC) model, proinflammatory cytokines can induce acute and chronic levels of inflammation associated with SCI. The different types of glycans and their level of expression involved in the MGC model were further considered from the acute to the chronic stages of inflammation. Lectin microarray and lectin immunostaining were also carried out to identify glycans associated with mixed glial cells and their modulation under treatments. Notably, an attempt was made to identify overall glycosylation (O‐linked and N‐linked), providing insight into a new glycotherapeutic approach for understanding inflammation and glial scar formation. This phase of the study hypothesizes that a cytokine‐induced inflamed MGC in vitro model will show differential glycans from the acute to the chronic phase of inflammation associated with SCI. To investigate this hypothesis, this study included the following objectives: study the expression pattern of glycans in MGC under an inflammatory a cytokine combination and LPS treatments using lectin microarray technique; validate the glycan expression in mixed glia under a cytokine combination and LPS treatments using lectin staining. The effect of sialyltransferase inhibitor (STI) on sialylation expression and mitochondrial function was studied in a mixed glial culture model in an inflammatory environment to assess the efficacy of addressing glycosylation profile alterations. To the best of the authors' knowledge, this is the first study to show that glycosylation inhibitors can replace physiological glycosylation in mixed glial culture model cells and that glycosylation plays a role in neuroinflammation. In the future, this could provide new molecular targets for biomaterial functionalization.

## 2 MATERIALS AND METHODS

### 2.1 Cell culture

As previously described, primary mixed glial cultures (MGCs) were prepared from spinal cords.^24^ Spinal cords were isolated by hydraulic extrusion technique from three‐day‐old post‐natal rats with minor modification in the spinal cord extrusion method.^25^ Later, meninges were gently peeled from spinal cords under microdissection microscope. Spinal tissues were chopped into fine (approx. 1 mm) pieces and digested using 1% trypsin‐Ethylenediaminetetraacetic acid (EDTA) solution for 15–20 minutes. Trypsin's activity was inhibited using Dulbecco's Modified Eagle Medium (DMEM)‐high glucose supplemented with 10% fetal bovine serum (FBS) and 1% Penicillin/Streptomycin (P/S). Further, MGCs were cultured as previously described (reference). The MGCs were grown in vitro for 3 weeks before treatments were applied. Early passage number was maintained throughout all experiments.

### 2.2 Western blotting

Cells of density 5 × 10^5^ cells/ml seeded into 6‐well plates and after respective treatments, cells were lysed in a radioimmunoprecipitation assay (RIPA) buffer (50 mmol/L Tris–HCl, pH 8.0, 150 mmol/L NaCl, 0.02% sodium azide, 0.1% sodium dodecyl sulphate [SDS], 1% Nonidet P‐40, 0.5% sodium deoxycholate) (Sigma‐Aldrich®, R0278) with protease inhibitor cocktail (1:100) (cOmplete™, EDTA‐free, Roche, Inc., 11873580001), phenylmethylsulfonylfluoride (1:50) (Sigma‐Aldrich®, 93482) and phosphatase inhibitor cocktail (1:10) (PhosSTOP™, Roche, Inc., 04906845001). The protein concentration in the total cell lysate was determined using a BCA protein assay kit (Pierce™ Bicinchoninic acid [BCA] Protein Assay Kit, Thermo Fischer Scientific™, 23225). An equal amount of protein from each sample was separated by 10%–12% sodium dodecyl sulfate (SDS) polyacrylamide gel electrophoresis and transferred to Hybond® ECL™ nitrocellulose membrane. The membrane was blocked with either 5% milk or 5% bovine serum albumin (BSA) depending upon the suitability of primary antibodies to avoid non‐specific binding of antibodies. This was followed by primary antibody incubation overnight at 4°C with 1:2000 rabbit anti‐P‐NFκB‐p65 (Ser536) (93H1) (Cell signaling, 3033s), mouse anti‐ NFκB‐p65(F‐6) (Santacruz biotech, sc‐8008) or 1 h at room temperature (RT) with 1:15,000 anti‐β‐actin (Sigma, A5441) on a rocking platform. All the washing steps were carried out in Tween‐20 in TBS (0.1%). Next, horseradish peroxidase‐conjugated secondary goat anti‐ rabbit or goat anti‐mouse antibodies (prepared in 5% milk or 5% BSA at 1:10,000) were applied followed by enhanced chemiluminescence detection. Signals from protein bands were captured on X‐ray films (CL‐XPosure™ Film, Thermo Scientific™, 34090) which were further analyzed using Image Studio™ Lite software and signals recorded as pixel density.

### 2.3 Cell protein extraction and glycoprotein sample labelling

**FIGURE 1:**
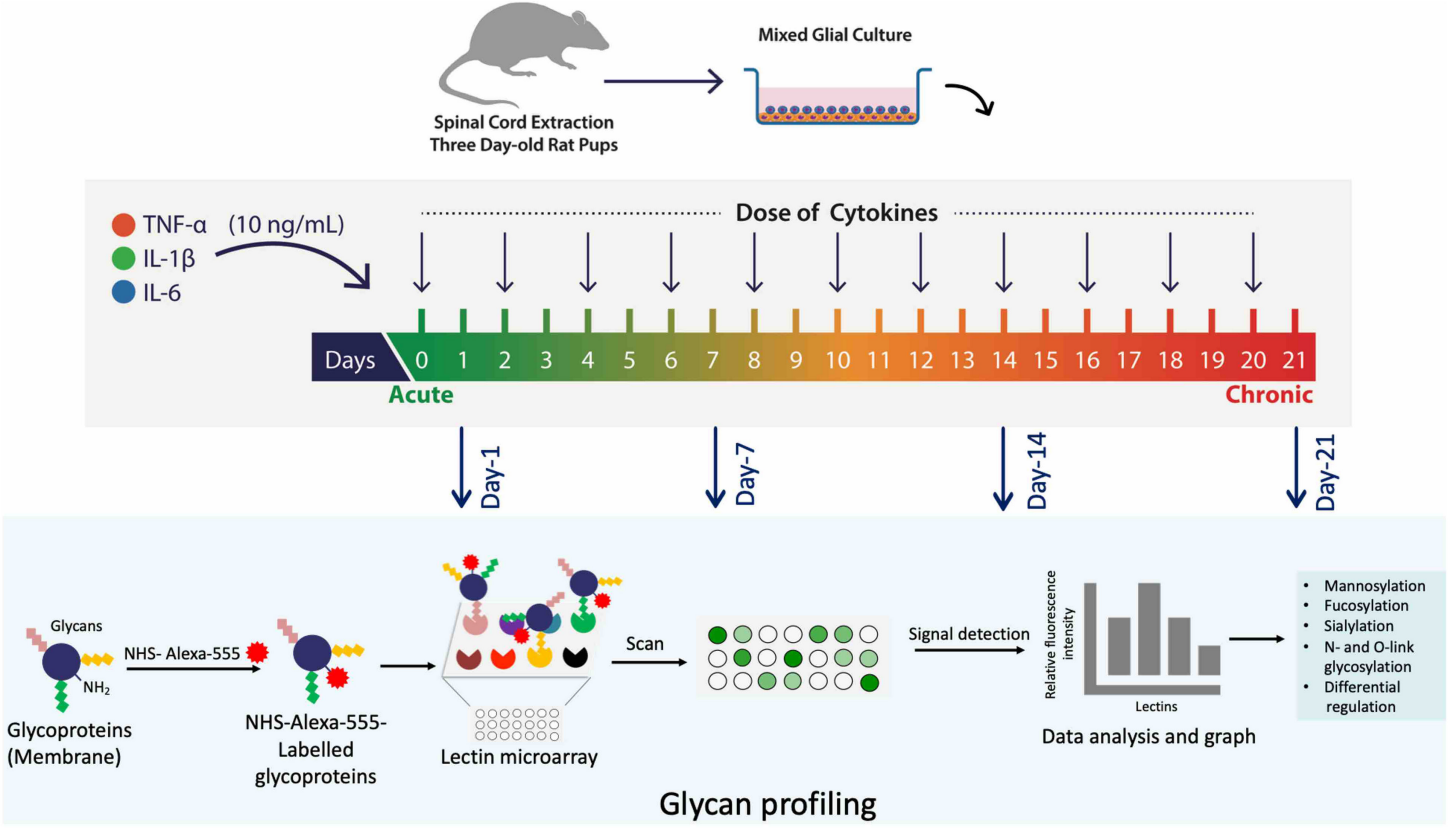
Workflow describing steps involved in the lectin microarray. MGCs were prepared from spinal cords isolated by the hydraulic extrusion technique from three‐day‐old post‐natal rats. MGCs were treated with TNF‐α, IL‐1β and IL‐6 (10 ng/ml per cytokine) in combination/s for 1 day (acute) to 21 days (chronic). Lipopolysaccharide (LPS) (10 ng/ml) was used as positive control. Treatments were given every alternate day (*n* = 3). Membrane proteins were extracted using Mem‐PER™ Plus, a membrane protein extraction kit. Lectin microarray profiling was performed using a panel of 48 lectins to study any alteration in mammalian glycosylation. MGCs were then assessed for glycosylation localisation and association with particular cell types using FITC‐conjugated lectins and glial markers (GFAP and CD11b), respectively, by ICC

As depicted in Figure 1, MGCs were seeded at a density of 1 × 10^5^ cells/well on PLL‐coated 24‐well plates with media (DMEM + 1% P/S + 10% FBS). After growing in an incubator for 2 days, the cytokine treatment was given for 21 days as described in Ref. [4]. Cells were treated with a combination of 10 ng/ml of three cytokines (TNF‐α, IL‐1β, and IL‐6) (R & D Systems; Recombinant rat TNF‐alpha protein, 510‐RT‐010; Recombinant rat IL‐1 beta/ IL‐1F2 protein, 501‐RL‐010; Recombinant rat IL‐6 protein, 506‐RL‐010) at day 0 and every subsequent 2 days (i.e., day 0, 2, 4, 6, 8, 10, 12, 14, 16, 18 and 20) until day 21. At each of the treatment time‐points (except day 0), half of the media was changed and filled with fresh cytokine treatment media. An LPS treatment group underwent the same procedure, with the cytokines substituted by a 100 ng/ml dose of LPS (Sigma Aldrich). The ctrl (control) group was carried out by performing the same half‐media change procedure with low‐serum media containing no added inflammatory molecules.

Samples of each group were extracted from the culture on days 1, 7, 14, and 21. A membrane Protein Extraction kit (Mem‐PER™ Plus, 89842) from Thermo Scientific was used to extract samples from cells (adherent mammalian cells). In short, after media removal cells were scraped and washed with cell wash solution. Harvested cells were permeabilised using permeabilization buffer, centrifuged, and the supernatant was collected (as it contains cytosolic proteins) and stored at −80°C. The pellet was resuspended with Solubilization Buffer, incubated, centrifuged, and the supernatant was collected as it contains membrane proteins and stored at −80°C. The membrane protein fraction would be further used for lectin micro‐array.

10 μl of phosphate buffer 10× (500 mmol/L, pH = 8.3) was added to the glycoprotein solutions in PBS (100 μl). Afterwards, the solution was treated with Alexa‐555‐N‐Hydroxysuccinimide (NHS) dye 1 μg/μl in Dimethyl Sulfoxide (DMSO) for 1 h at room temperature (RT) in such a way that 0.15 μl of dye was employed to label 1 μg of glycoprotein. The addition of Tris buffer quenched the excess of dye. The fluorescently labeled glycoprotein solutions were not purified and were directly used in the lectin array analysis.

### 2.4 Lectin microarray construction, incubation, and data extraction

Lectin solutions 0.4–0.5 mg/ml and antibody solutions 0.1 mg/ml, were prepared in print buffer (1.0 mmol/L D‐glucose in PBS containing 0.01% of cy3‐conjugated BSA). Aliquots of 20 μl volume of each freshly prepared lectin solution were loaded in a 384 well plate. Volumes (0.67 nl) of these dilutions were spotted onto an N‐Hydroxysuccinimide (NHS) functionalized glass slide in 6 replicates. The humidity in the print chamber was maintained at around 50% before and during printing. The slides containing the lectin arrays were incubated after printing in a 75% humidity chamber at 18°C overnight and stored at 20°C without quenching if not used immediately. The remaining NHS groups were quenched by placing the slides in a 30 mmol/L ethanolamine solution in borate buffer for 1 h and blocked with a 0.3 mg/ml bovine serum albumin (BSA), 0.3 mmol/L Ca^2+^ solution in phosphate‐buffered saline (PBS) buffer containing 0.5% Tween‐20 for 1 h.

Printed slides, stored at −20°C, were quenched (NHS‐activated hydrogel coated glass surface) by immersion in a 50 mmol/L ethanolamine solution in borate buffer (50 mmol/L, pH 8.5) for 30 min at RT. The quenched surface was then passivated by incubation in PBS buffer containing 0.5% Tween‐20, BSA 0.4 mg/ml, CaCl2 0.3 mmol/L, for 30 min at RT. The glycoprotein samples were added to the corresponding wells, and the incubation was carried out for 1.5 h at RT. Then the glycoprotein solutions were removed, and the slide washed with PBS for five mins and dried by centrifugation and scanned. The images obtained from the G265BA microarray scanner (Agilent Technologies) were analyzed with Pro ScanArray Express™ software (PerkinElmer). Samples were incubated in the lectin array at a concentration of 3 μg/ml. The dilution of the samples was performed by the addition of lectin incubation buffer to the labeled glycoprotein solutions after labelling. The labeled‐BSA and control dye sample was incubated under the same concentration conditions as negative controls for lectin binding. Additionally, ‘mean pixel density’ from each lectin was calculated, and the data plotted as a heatmap. Hierarchical clustering of the data was performed using Morpheus, https://software.broadinstitute.org/morpheus.

### 2.5 Lectin cytochemistry

High‐grade bovine serum albumin (BSA; Sigma, A7638) was treated with periodic acid (Sigma, P0430) before its use for blocking according to an established protocol.^26^ Briefly, 5 g of BSA was dissolved in 100 ml of freshly made periodic acid solution (10 mmol/L periodic acid in 0.1 M sodium acetate, pH 4.5) and incubated at room temperature for 6 h. The BSA was dialyzed against the water with four water changes over 2 days at 4°C and finally lyophilized and stored at 4°C. The cytochemistry procedure was followed as discussed^27^ with few modifications for staining on cells. First, the cell media was removed, and the cells were washed twice with 1× Tris‐buffered saline (TBS) (10 mmol/L Trizma hydrochloride, 0.1 M NaCl, 1 mmol/L CaCl2, 1 mmol/L MgCl2, pH 7.2). Cells were then fixed in 4% PFA for 15 min, washed four times with TBS, and then blocked with 2% periodic acid‐treated BSA in TBS for 30 min at room temperature. The cells were incubated with the appropriate concentration of fluorescein isothiocyanate (FITC)‐labeled lectins (EY Laboratories Inc., San Mateo CA) in the dark for 1 h at RT at the following concentrations (Table S2): Phaseolus vulgaris erythroagglutinin (PHA‐E) and Ricinus communis (RCA)‐I at 10 μg/ml, Sambucus nigra (SNA)‐I, Peanut agglutinin (PNA), Wheat germ agglutinin (WGA), Maackia amurensis agglutinin (MAA), Galanthus nivalis agglutinin (GNA), Datura stramonium (DSL) and Ulex europaea agglutinin (UEA)‐I at 20 μg/ml, and Wisteria floribunda agglutinin (WFA) at 30 μg/ml. Negative controls were carried out in parallel with a TBS solution containing no lectins (Figure S8). Haptenic sugar inhibition controls were carried out in parallel by co‐incubation of 100 mmol/L appropriate sugar in TBS with the following lectins as follows (Table S2): SNA‐I, WFA, and PNA were prepared in lactose, WGA and GNA in mannose, MAA and RCA‐I in galactose, PHA‐E in bovine IgG, DSL in N‐Acetyl‐D‐glucosamine (GlcNAc) and UEA‐I in fucose (all carbohydrates, Sigma Aldrich). After lectin staining, a traditional immunocytochemistry procedure was performed to label astrocytes (GFAP) and microglia (CD11b).

### 2.6 Immunocytochemistry

After lectin cytochemistry, cells were permeabilized by incubating in 0.2% Triton X‐100 in PBS and washed with PBS. Unspecific bindings were blocked with 1% BSA+ 10% NGS in PBS solution (blocking buffer) for 1 h at RT. Primary antibodies, anti‐GFAP (1:500, Dako, Z033429), anti‐CD11b (1:200 Sigma, CBL1512) were prepared in blocking buffer and incubated overnight at 4°C. Incubated with secondary antibody Alexa Fluor® 488 (1:500, Thermo Scientific™, A‐10667) and Alexa Fluor® 546 (1:500, Thermo Scientific™, A11035) for 1 h at RT. Coverslips were mounted on slides by placing a small drop of mounting medium containing DAPI (Fluoromount‐G™, Thermo Scientific™, 00‐4959) on the slide and placing the coverslip cell‐surface down.

### 2.7 Microscopy and image analysis

Fluorescent cytochemistry images were captured on an Olympus VS120 Virtual Slide Microscope with Olympus VS fluorescence software (VS‐ASW‐FL). Every image was taken at the same exposure fluorescence intensity to ensure consistency. Cell morphology and lectin‐localization were qualitatively assessed for each sample. Additionally, lectin staining fluorescence intensity (FI) was quantitatively analyzed using the ImageJ (Fiji) software. The number of Hoechst‐positive nuclei was quantified. Fluorescence intensity per cell (FIPC) was calculated by dividing the FI by the number of Hoechst‐stained nuclei per image. FIPC was measured for each sample (*n* = 3), for each lectin staining (SNA‐I, WFA, PNA, WGA, MAA, PHA‐E, UEA‐I, GNA, DSL and RCA‐I), for each group (control, cytokine‐treated and LPS treated) at 24 h.

### 2.8 Seahorse Cell Mito Stress assay

The assay was performed as explained in Ref. [4] 40,000 cells/well were seeded into the XFp Seahorse miniplates. After growing in an incubator for 2 days in vitro (DIV), the media was replaced with (DMEM +1% P/S) and cells were treated with a combination of pro‐inflammatory cytokines (TNF‐α, IL‐1β, and IL‐6) of dose 10 ng/ml (per cytokine) and STI (Sialyltransferase Inhibitor, 3Fax‐Peracetyl Neu5Ac) (300 μmol/L), 566224, Sigma‐Aldrich at day 0. On the day of assay, the media was replaced with glucose, pyruvate, L‐glutamine and phenol red‐free Seahorse XF base medium. Assay medium was prepared with the addition of glucose (25 mmol/L), sodium pyruvate (1.0 mmol/L) and L‐glutamine (2.0 mmol/L). The pH was adjusted using 1 N NaOH to 7.4 then media filtered through a 0.2 mm filter. Before the assay, the sensor cartridge was hydrated overnight, and seahorse instrument was turned on at least 5 h before the assay. Oxygen consumption rate (OCR) and extracellular acidification rate (ECAR) was measured on XFp Seahorse analyzer with the sequential addition of 1. Oligomycin (1 μmol/L), 2. FCCP (2 μmol/L), 3. Rotenone/antimycin (0.5 μmol/L) for 110 min with five readings per cycle.

After assay was finished, cells were lysed in a radioimmunoprecipitation assay (RIPA) buffer (50 mmol/L Tris–HCl, pH 8.0, 150 mmol/L NaCl, 0.02% sodium azide, 0.1% sodium dodecyl sulphate (SDS), 1% Nonidet P‐40, 0.5% sodium deoxycholate) (Sigma‐Aldrich®, R0278) with protease inhibitor cocktail (1:100) (cOmplete™, EDTA‐free, Roche, Inc., 11873580001), phenylmethylsulfonylfluoride (PMSF) (1:50) (Sigma‐Aldrich®, 93482) and phosphatase inhibitor cocktail (1:10) (PhosSTOP™, Roche, Inc., 04906845001). The protein concentration was determined using a BCA protein assay kit (Pierce™ BCA Protein Assay Kit, Thermo Scientific™, 23225). OCR and ECAR data were normalized on Wave software using protein concentration per well. The extracellular acidification and rate of ATP production by glycolysis and oxidation were calculated using ECAR and OCR values.^28^

### 2.9 Statistical analysis

All statistical analyses were performed using GraphPad Prism 8.00 software. We performed a Shapiro–Wilk test of normality to assess data distribution. Data that have exhibited a normal/Gaussian distribution have been analyzed using one‐way analysis of variance (ANOVA). It was followed by Tukey multiple comparison test for comparing more than three samples, and two‐tailed unpaired *t*‐tests for comparing two samples with 95% confidence. *p* < 0.05 was considered to be statistically significant.

## 3 RESULTS

The study aimed to decipher glycan modulation in glial cells involved during neuroinflammation associated with SCI. However, similar outputs could be liked with other neuroinflammatory phenomena. The study was divided into various levels to understand glycosylation in astrocytes and microglia in mixed glial cultures. Also, we focussed our approach to show the effect of sialyltransferase inhibitor on sialic acid expression in MGC and mitochondrial respiration.

### 3.1 Study of lectin microarray upon cytokine combination treatment from the acute to the chronic phase of inflammation

During glial activation, glycans are significantly expressed and modulate an immune response. Therefore, it was essential to analyze the type of glycans involved and their correlation with cell types in MGC from the acute to the chronic phase. To understand this, the membrane proteins were extracted after the treatments at days 1, 7, 14 and 21 from MGC. Each sample with glycoproteins was tagged with Alexa‐555, and 3 ug/mL of it was incubated with a lectin microarray panel of 48 lectins (Table S1). The signal intensity was detected to quantify the amount of glycoprotein binding with the respective lectin (Figure 1).

**FIGURE 2:**
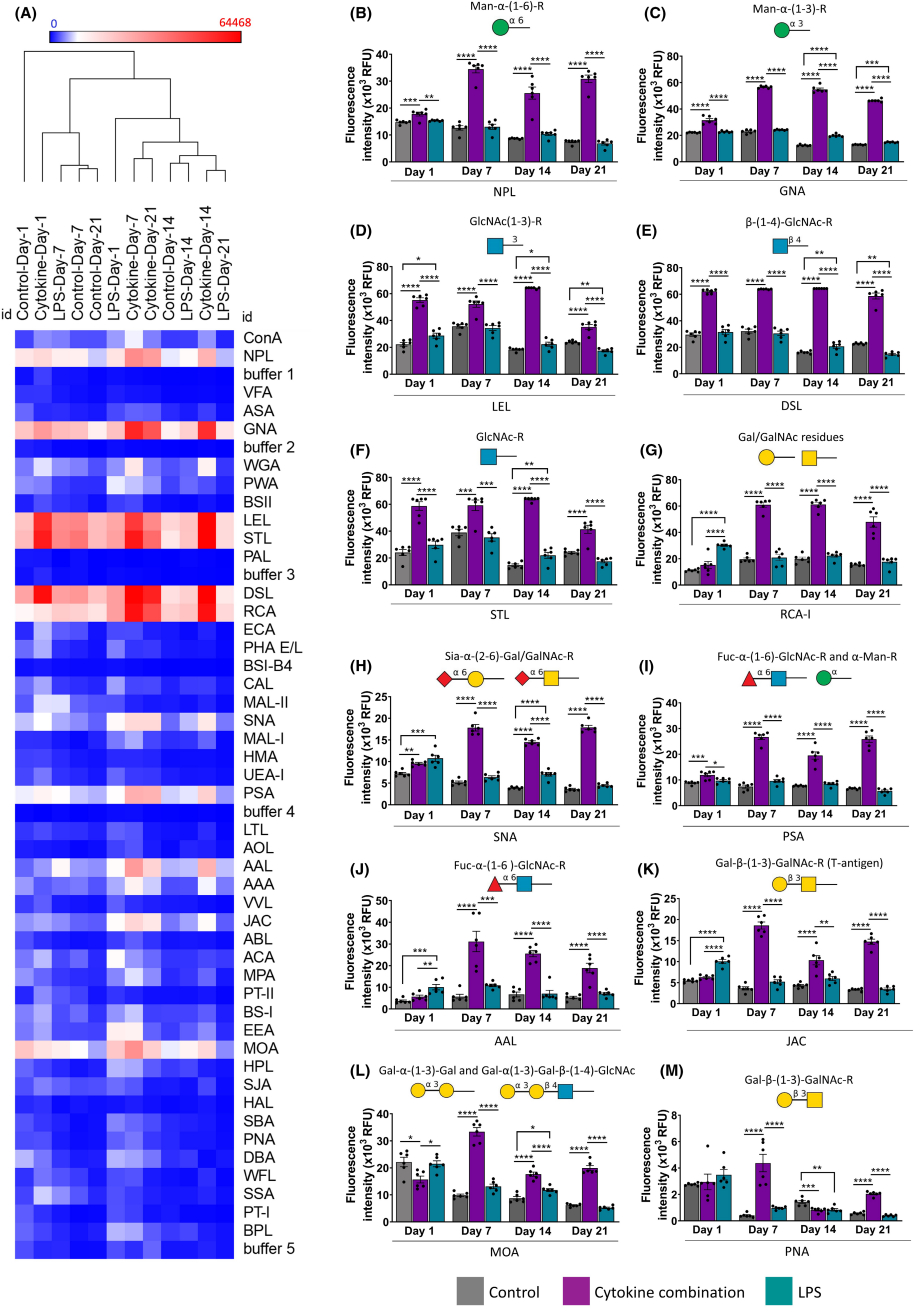
Identification of glycan modulations during cytokine combination and LPS treatments. (A) Hierarchical clustering analysis of 48 lectins. Colors define activation as highly expressed (red) and no expression (blue). Only one cytokine combination (i.e., TNF‐α, IL‐1β and IL‐6 combination) was used along with LPS as a positive control. Treatment was given from day‐1 up to day‐21, and at four time points, day‐1, day‐7, day‐14 and day‐21 lectin microarray was performed on membrane proteins. Lectin microarray interaction pattern of control, at day 1, day 7, day 14 and day 21. Alexa‐555‐NHS dye tagged membrane glycoproteins were printed on an NHS‐activated hydrogel‐coated glass surface. Samples at a 3 μg/ml concentration were incubated in the lectin array. Images were obtained by G265BA microarray scanner (Agilent Technologies) and were analyzed with Pro Scan Array Express™ software (PerkinElmer). Labeled‐BSA and control dye samples were incubated at the same concentration conditions as negative lectin binding controls. The cytokine combination group showed an increased signal for the lectins with a higher response. In (B) Narcissus Pseudonarcissus (NPL), (C) Galanthus nivalis agglutinin (GNA), (D) Lycopersicon esculentum (LEL), (E) Datura stramonium (DSL), (F) Solanum tuberosum (STL), (G) Ricinus communis agglutinin‐I (RCA‐I), (H) Sambucus nigra (SNA), (I) Pisum sativum (PSA), (J) Aleuria aurantia lectin (AAL), (K) Jacalin (JAC), (L) Marasmium oreades agglutinin (MOA) and (M) Peanut agglutinin (PNA) (days 1, 7, 14 and 21), indicating higher general glycosylation upon cytokine combination treatment. Data are represented as mean ± SD, *n* = three independent experiments pulled samples run for six technical replicates, *p < 0.05, **p < 0.01, ***p < 0.001 and ****p < 0.0001. Man: Mannose, GlcNAc: N‐acetylgalactosamine, Gal: Galactose, LacNAc: N‐acetyl lactosamine, Sial: Sialic acid, Fuc: Fucose, α‐Gal: α‐Galactose

Pearson's correlation hierarchical clustering metric analysis was performed to identify trends in the treatment groups. As depicted in Figure 2A, the groups were separated into several clusters, illustrating the heterogenicity among the control, cytokine combination, and LPS treated groups and the day on which treatment was given. There was a clear distinction between cytokine combination and LPS treated samples compared to the control group. The treatment groups in early time points (day one and seven) were separated into different clusters than later time points (day 14 and 21). As mentioned in the heatmap, several lectins such as NPL, GNA, LEL, STL, DSL, RCA‐I, SNA, PSA, AAL, JAC and MOA showed strong binding. Therefore further analysis was carried out to show their precise expressions.

The dye‐tagged glycoproteins showed a strong interaction with mannose binding lectins such as NPL and GNA, revealing mannoside N‐glycans and/or O‐mannosylation (Figure 2B,C). MGCs also present a firm binding with N‐acetyl glucosamine (GlcNAc) binding lectins such as LEL and STL, which reported the presence of multi‐antennary N‐glycans (Figure 2D,F). Further, the DSL lectin interaction showed the potential poly‐N‐acetyl lactosamine (LacNAc) epitope on the glial membrane (Figure 2E). The membrane glycoproteins showed binding to galactose lectins such as RCA‐I, which interacts with terminal beta‐galactose residues (Figure 2G). There was a low interaction with ECA, which can be interpreted as a low amount of terminal N‐acetyl galactosamine (GalNAc) (Figure 2A).

Regarding sialylation, the samples interacted with SNA lectin, reporting 2,6‐sialylation, whereas the signal for MAL‐I, which interacts with 2,3‐sialic acid, was considerably lower (1.34‐fold, *p* < 0.01) at day one. However, on days 7, 14 and 21 the interaction was higher in the cytokine‐combination treated group (3.45–4.8‐fold, *p* < 0.0001) (Figure 2A,H). Interaction with fucose‐binding lectins was also crucial with PSA, showing fucosylated biantennary N‐glycans and/or high mannose glycans (Figure 2I), AAL and AAA, which showed the presence of fucose, mainly 1,6‐fucosylation (Figure 2A,J). There was also significant binding to T‐antigen and α‐Gal lectins, indicating the potential presence of O‐glycans. The lectins JAC, ACA and MPA show the presence of T‐antigen, Gal‐β‐(1‐3)‐GalNAcα O‐glycan epitopes (Figure 2A,K). The lectins MOA and EEA recognize O‐glycans with the Gal‐α‐1,3‐Gal epitope. Regarding GalNAc lectins, the sample showed a signal for them at each time point. However, the binding affinity was significantly reduced compared with others (3–10‐fold) (Figure 2A,L).

When treatments were grouped according to days (1, 7, 14, 21, and), it was observed that the signal had increased for the lectins with a higher response in the group of cytokine combination. This phenomenon was observed, for example, in the case of NPL, GNA, LEL, STL MOA, PSA, RCA‐I and DSL (days 1, 7, 14 and 21) and SNA, AAL, AAA, PNA and JAC (days 7, 14, 21), indicating higher (*p* < 0.0001) general glycosylation in the group mentioned above. Interestingly, the cytokine combination group showed more significant glycosylation profile (*p* < 0.0001) changes than control and LPS treated groups (Figure 2A).

### 3.2 Localisation of glycans on astrocytes and microglia

**FIGURE 3:**
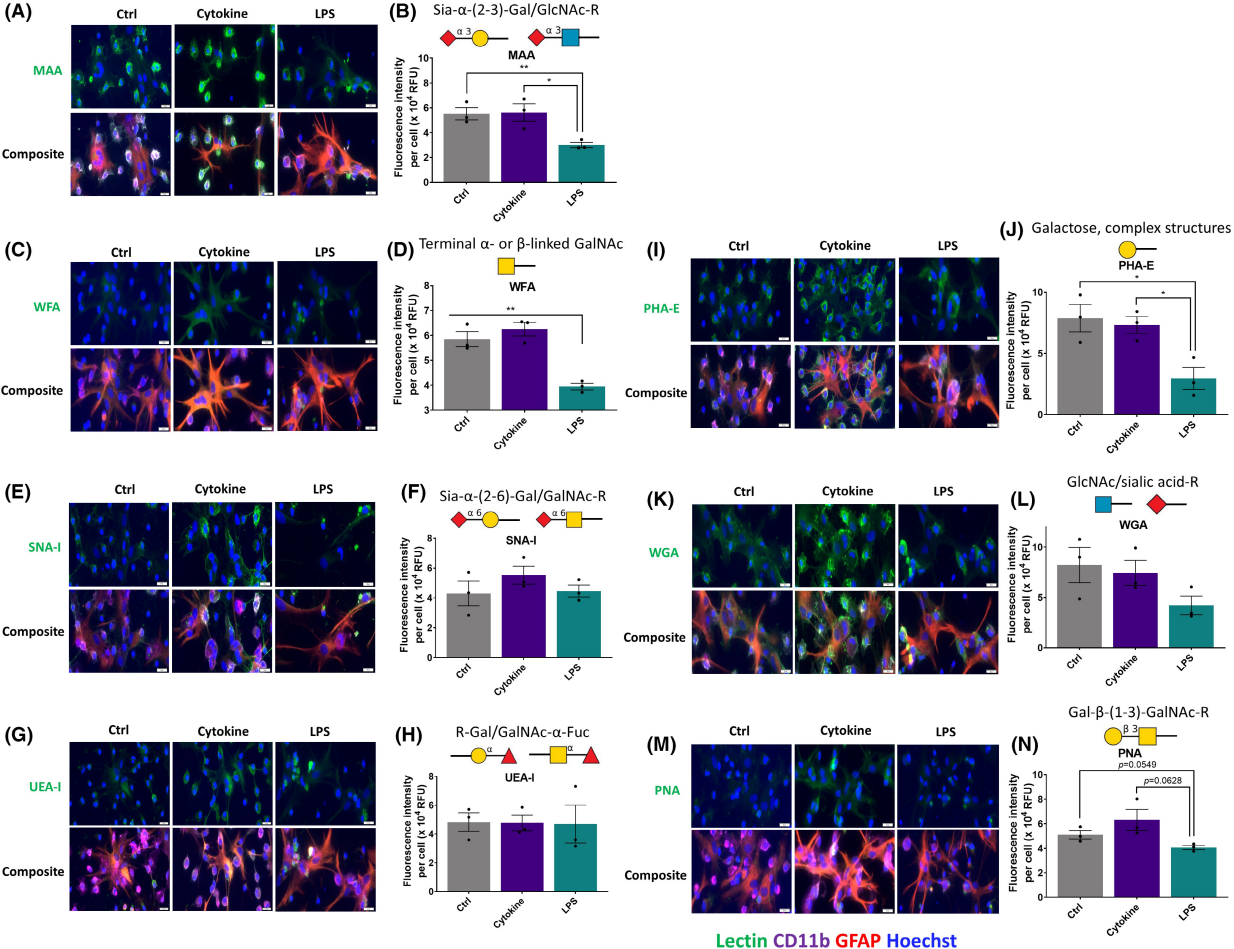
Effect of cytokine combination and LPS on the expression of lectins. Fluorescence intensity per cell was quantified and averaged for each lectin of the three treatment groups at 24 h. (A,B) MAA, (C,D) WFA, (I,J) PHA‐E and (M,N) PNA showed significant differences between groups. (E,F) SNA‐I, (G,H) UEA‐I and (K,L) WGA showed no significant differences between the three treatment groups. Individual staining of astrocytes and microglia has been shown in Figure S9. Data are represented as mean ± SEM, *n* = 3 three independent experiments, one‐way ANOVA followed by multiple comparisons Tukey post hoc test. *p < 0.05. Scale bar: 20 μm.

**FIGURE 4:**
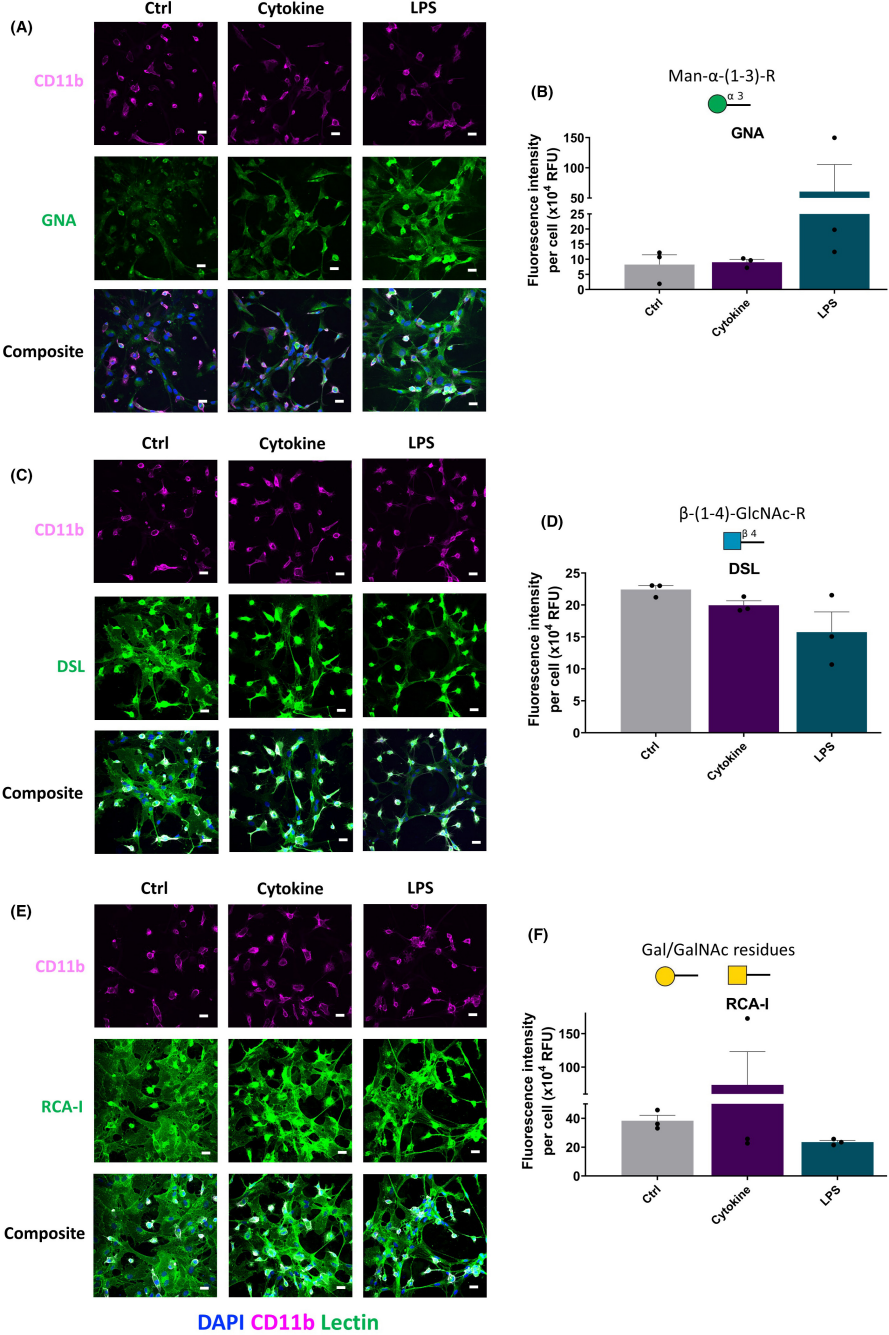
Effect of cytokine combination and LPS on the expression of glycans. Fluorescence intensity per cell was quantified and averaged for each lectin of the three treatment groups at 24 hrs. (A,B) GNA, (C,D) DSL, (E,F) RCA‐I showed no significant differences between the three treatment groups at 24 h. Data are represented as mean ± SEM, *n* = three independent experiments, one‐way ANOVA followed by multiple comparison Tukey post hoc test. Scale bar: 20 μm

After the lectin microarray, lectin immunostaining was performed to identify the glycans' localisation on glia and quantify the total lectins' total intensity (Figure S9). GNA lectin staining showed expression of α‐1,3 mannose on mixed glia, and the LPS treated group showed higher expression at day one (7.34‐fold) (Figure 4A,B). PHA‐E staining, which identifies GlcNAc residues, did not display any difference between control and cytokine‐ combination‐treated samples; however, LPS treatment reduced expression level (*p* < 0.05) (Figure 3I–J).

Upon performing DSL lectin staining (which binds β‐(1–3)‐GlcNAc residues), it was observed that β‐(1–3)‐GlcNAc residues were associated with mixed glia, and there was no significant difference between control, cytokine combination and LPS treated groups (Figure 4C,D). Upon lectin ICC, RCA‐I's binding with GalNAc on mixed glia was higher (1.92‐fold) in the cytokine‐combination‐treated group (Figure 4E,F). WGA lectin, which binds with β‐(1–4)‐GlcNAc residue, showed increased binding with astrocytes and microglia upon cytokine combination (0.91‐fold), whereas this decreased after LPS treatment (WGA, 1.95‐fold) (Figure 3K,L). PNA, which binds with β‐(1‐3)‐GalNAc residue, showed increased binding with astrocytes and microglia upon cytokine combination (1.24‐fold), whereas this decreased after LPS treatment (*p* < 0.0549) (Figure 3M,N). Further, staining was also carried out for α or β‐GalNAc residues associated with chondroitin sulphate using WFA lectin. It was found that WFA binding was associated with astrocytes in the control and cytokine treated groups. However, LPS treatment showed lower WFA binding (*p* < 0.01) (Figure 3C,D).

Upon lectin staining, SNA‐I lectin showed more binding with α‐2,6‐sialic acid moieties of microglia than with astrocytes, and quantification showed a higher signal in the cytokine‐combination treated group (1.29‐fold). LPS did not show any change compared to the control group (Figure 3E,F). Additionally, MAA, which identifies α‐2,3‐sialyation, showed strong binding with microglia and upon LPS treatment the binding was reduced (*p* < 0.01) (Figure 3A,B). Lectin staining using UEA‐I lectin showed binding for fucosylation on both astrocytes and microglia (Figure 3G,H). The summary of lectin staining outputs is mentioned in Table S3.

### 3.3 Sialyltransferase Inhibitor, 3Fax‐Peracetyl Neu5Ac (STI) treatment downregulates the sialic acid expression

One glycans, sialic acid, is associated with the modulation of inflammation in various conditions. We found lectins binding with sialic acid linkages in lectin microarray, which was further confirmed by staining of MAA and SNA‐I. In neural tissue, sialic acid is predominantly present in the form of polysialic acid associated with the neural cell‐adhesion molecule (NCAM). In CNS N‐glycans are either or both sialylated and/or fucosylated, which governs a particular function of N‐glycans. Hypersialylation is a characteristic feature in which sialyltransferase (and other 36 enzymes) increases (polysialyltransferases). It catalyzes different sialic acid linkages such as α‐(2‐6, or, 2‐8) on glycan chains.^12^ Therefore, we decided to understand the effect of STI on sialic acid expression in MGC and metabolomics associated with it under normal and inflammatory conditions.

**FIGURE 5:**
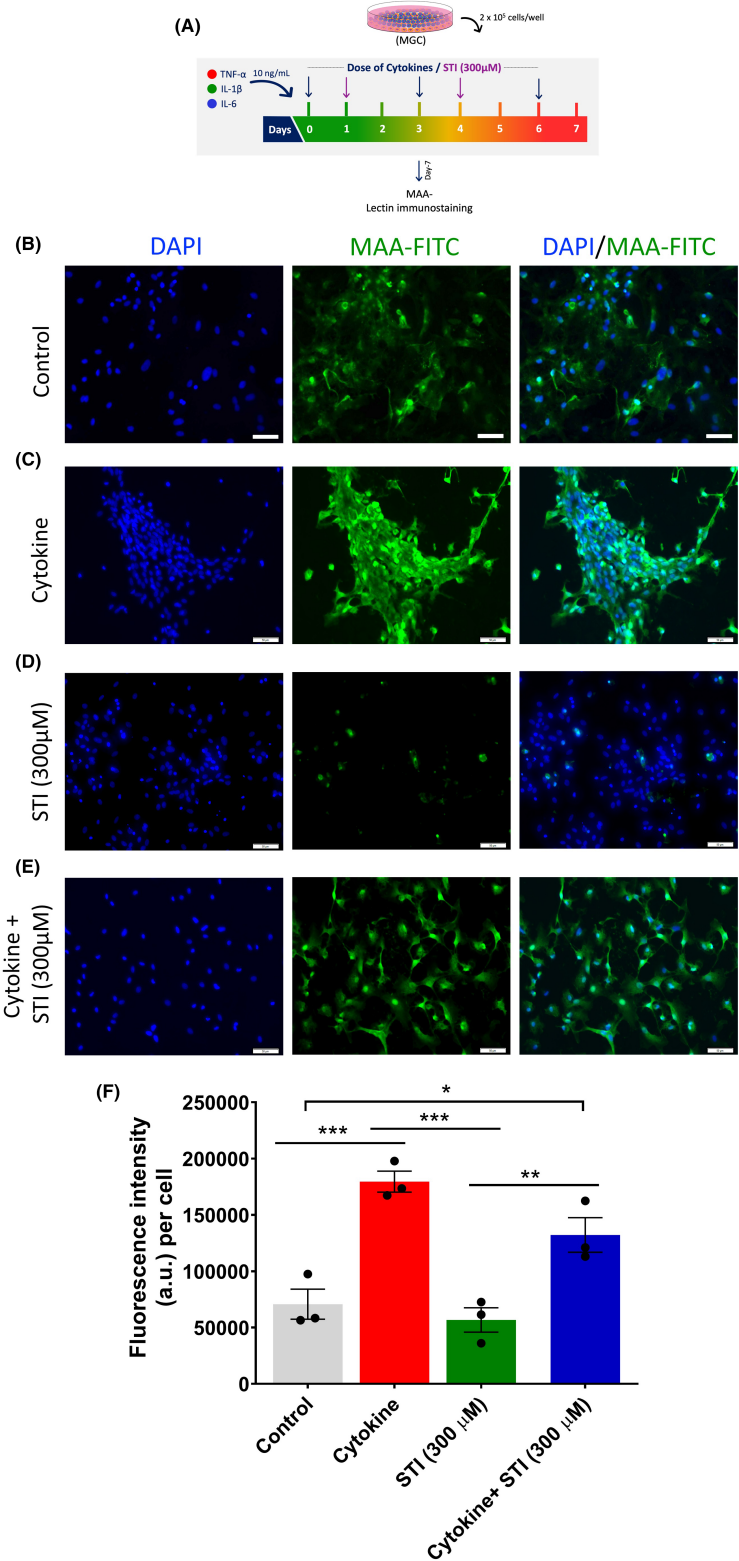
Effect of Sialyltransferase Inhibitor, 3Fax‐Peracetyl Neu5Ac (STI) on MAA lectin binding on MGC under cytokine combination treatment. (A) Experimental design: 2 × 10^5^ cells/ml of MGC were seeded in 24 well plates. After 48 h they were given a cytokine combination treatment and this treatment was repeated on day three and day six. Instead of cytokine combination treatment, FBS‐free DMEM media was added in the control group. Meanwhile, STI with 300 μmol/L concentration was given on day one and subsequently on day four. At the end of day seven, cells were stained with MAA lectin to see sialic acid expression. MAA recognizes α‐(2,3)‐linked sialic acid. (B) Control group, (C) Cytokine (cytokine combination), (D) STI (300 μmol/L), (E) Cytokine + STI (300 μmol/L) and (F) quantification of MAA binding fluorescence intensity (a. u.) per cell. Data are expressed as mean ± SEM, *n* = three independent experiments. One‐way ANOVA followed by multiple comparison Tukey post hoc test. *p < 0.05, **p < 0.01, ***p < 0.001. Scale bar: 50 μm

First we optimized the dose of the drug Sialyltransferase Inhibitor, 3Fax‐Peracetyl Neu5Ac (STI). We used three different concentrations 100, 200 and 300 μM. We treated it on MGC for 7 days (in which the dose was given on day 0 and day four). We used MAA lectin to identify α 2‐3‐linked sialic acid linkages based on reliable staining (Figure 3A) and significant upregulation in cytokine‐treated group (lectin microarray data) (Figure S1). Upon increase in the concentration, the binding affinity of MAA lectin reduced (Figure S2). We choose 300 μmol/L concentration for further use. We observed that even 3 days of treatment (in which dose was given on day 0) there was a decrease in MAA lectin binding (Figure S3). When we treated MGC for 7 days with cytokine combination treatment (Figure 5A), there was an increase in the MAA binding intensity in the cytokine combination treatment group, which was reduced upon STI treatment, which confirmed that STI could downregulate the higher expression of sialic acid during inflammation (Figure 5B–F). As NFkB‐p65 is a significant regulator in inflammation, we studied its relevance here. We performed western blotting to see the effect of STI on the NFkB‐p65 pathway in MGC under cytokine combination treatment. We found a higher expression of this pathway in all the groups treated in combination with cytokines compared to vehicle and STI only treated groups. However, there was no significant difference between cytokine only and cytokine with STI groups (Figure S6 and S7).

### 3.4 Sialyltransferase Inhibitor, 3Fax‐Peracetyl Neu5Ac (STI) treatment alters the mitochondrial function

**FIGURE 6:**
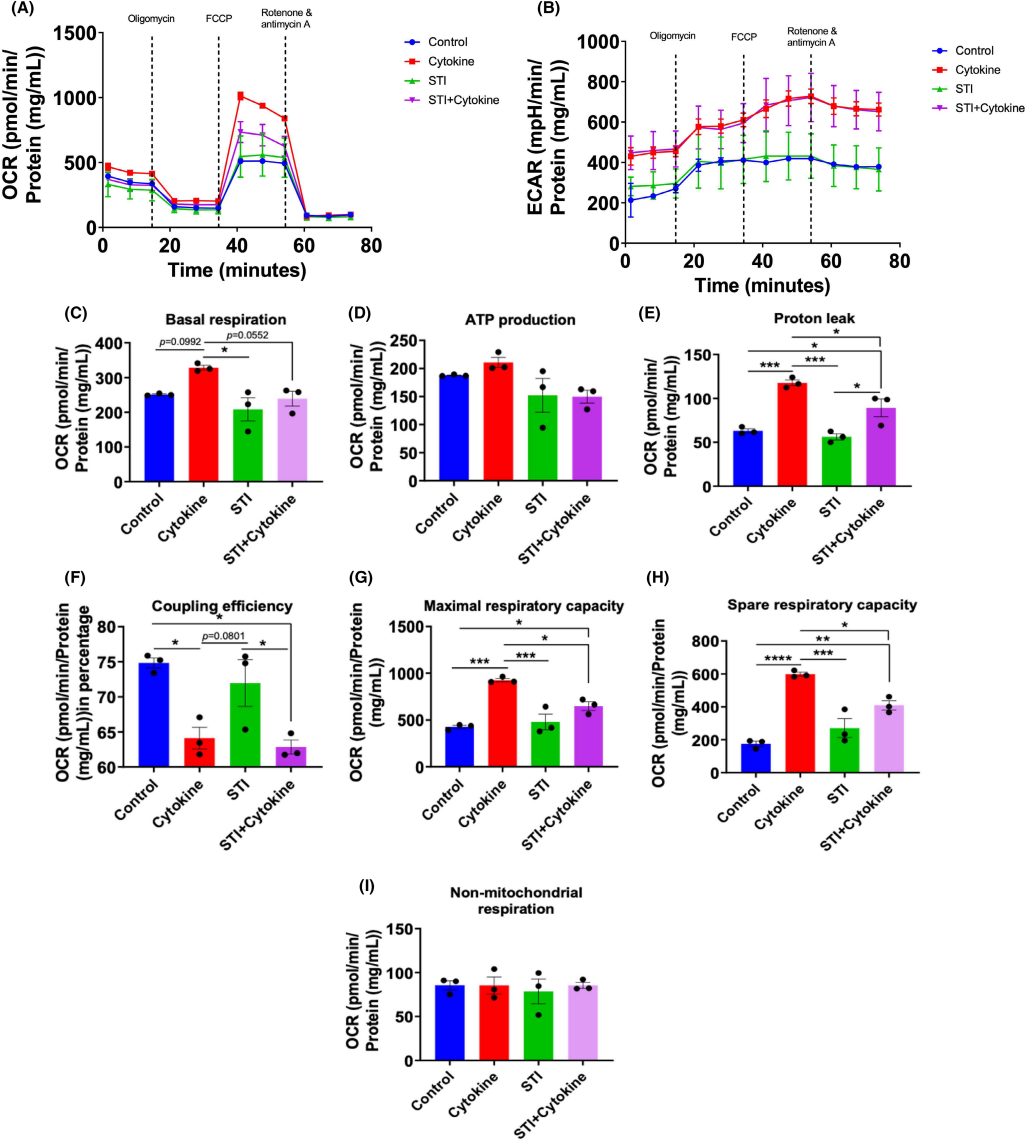
An STI (Sialyltransferase Inhibitor, 3Fax‐Peracetyl Neu5Ac) treatment reverts basal respiration, ATP production, proton leak, coupling efficiency, maximal respiratory capacity and spare respiratory capacity caused by cytokine treatment by day three. After 48 h, MGC cells were treated with cytokine combination, with or without STI at day 0 only, and on day three, cells underwent Cell Mito Stress assay. (A) Sequentially, OCR after adding three drugs (i.e. oligomycin, FCCP and Rotenone and antimycin A). (B) Extracellular acidification rate (ECAR) after adding the above mentioned three drugs sequentially. (C–I) All parameters were calculated as a cytokine combination and STI treatment function. For this, total protein per well was calculated using a BCA protein quantification assay and data was normalized against it. Seven parameters, namely, basal respiration, ATP production, proton leak, coupling efficiency, maximal respiratory capacity, spare respiratory capacity and non‐mitochondrial respiration, were measured and plotted as a bar graph. Data are represented as mean ± SEM, *n* = 3. *p < 0.05, **p < 0.01, ***p < 0.001, ****p < 0.0001. One‐way ANOVA followed by multiple comparison Tukey post hoc test was performed.

Seahorse Mito Stress assay was performed on day one and day three to assess mitochondrial function in MGC when treated with STI and/or cytokine combination. Oxygen consumption rate (OCR) and extracellular acidification rate (ECAR) were measured with the sequential addition of 1. Oligomycin (1 μmol/L), 2. FCCP (2 μmol/L) and 3. Rotenone/antimycin (0.5 μmol/L). We observed a decrease in the OCR and ECAR in the cytokine‐treated group at day one (Figure S4) but increased at day three (Figure 6). This is due to the activation of the glial cells. We used OCR values to determine mitochondrial activity. To understand bioenergetics phenotypes OCR and ECAR values were used to calculate the proton production rate (PPR) and rate of ATP production by glycolysis and oxidation.

**FIGURE 7:**
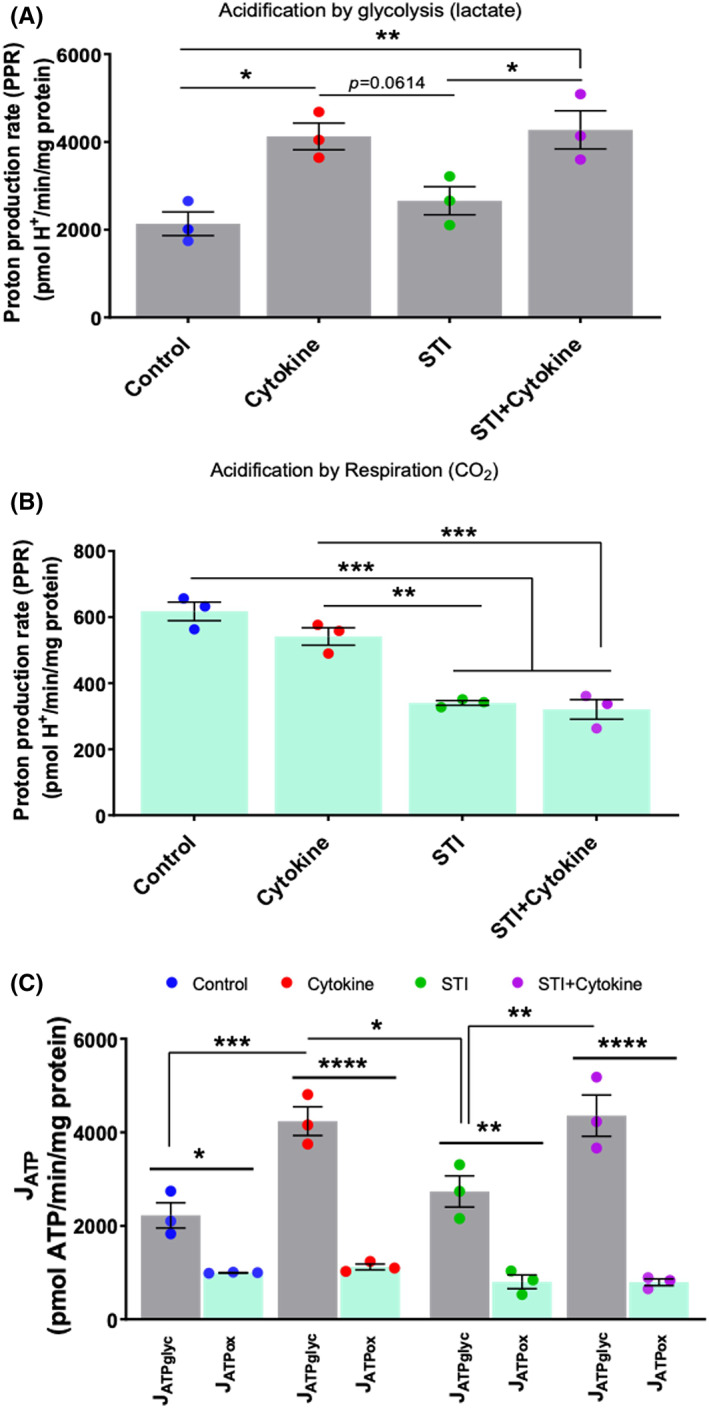
Bioenergetics phenotype of mitochondrial function (at day three). After 48 h, MGC cells were treated with cytokine combination, with or without STI at day 0 only and on day three, cells underwent Cell Mito Stress assay. (A) The rate of extracellular acidification is caused by glycolysis due to lactate production. Both the cytokine and the STI + cytokine treatment groups showed increased acidification by glycolysis compared to the control and the STI group. (B) The rate of extracellular acidification due to respiration by CO_2_ production. It was higher in the control and cytokine treated groups compared to the STI, and STI + cytokine treated groups. However, there was no difference in acidification by respiration between the STI and the STI + cytokine treatment group. (C) Data from basal ECAR and OCR has been converted to the rate of ATP production by glycolysis and oxidation using formula. The rate of ATP production by glycolysis was higher in the cytokine and the STI + cytokine treatment groups than in the control and the STI group (J_ATPglyc_). Under four treatments, the rate of ATP production by glycolysis was higher than that of ATP production by oxidation. Data are represented as mean ± SEM, *n* = 3. *p < 0.05, **p < 0.01, ***p < 0.001, ****p < 0.0001. One‐way ANOVA followed by multiple comparison Tukey post hoc test was performed.

The first parameter that we calculated was basal respiration. We found that control and STI treated groups had lower basal respiration than the cytokine treated group at day three (Figure 6A). Whereas upon STI treatment, it was decreased even in combination with cytokine at days one and three (Figure 6C and Figure S4C). This signifies that STI reduced activation of the glial cells, and it affected mitochondrial activity. This can be correlated with the acidification by oxidation parameter, as we observed STI was able to reduce the acidification by oxidation (Figure 7B). Similar outputs were observed with ATP production as basal respiration is directly linked with ATP production (Figure 6D and Figure S4D, Data S1).

Further analysis showed that at day one there was no significant difference between the rate of ATP production by glycolysis in cytokine treated groups and the rate of ATP production by oxidation (Figure S5). Whereas at day three the rate of ATP production by glycolysis was higher in cytokine treated groups compared to rate of ATP production by oxidation (Figure 7C). Later, the proton leak, which is a parameter often considered a hallmark of mitochondrial damage, was calculated and found higher in the cytokine group. It was significantly reduced upon STI treatment on day three (Figure 6E). In contrast, these two groups' coupling efficiency was lower than the control, and STI only treated the group on day three (Figure 6F). Which was expected as cytokine treatment imbalances the mitochondrial ability to couple the number of protons and the amount of ATP produced. The FCCP dependent maximal respiration (Figure 6G) and spare respiratory capacity (Figure 6H) was higher in the cytokine treated group. It was reduced upon STI treatment, which confirms that STI has an overall effect on astrocytes and microglia to lower their activation.

## 4 DISCUSSION

The inflammation associated with SCI is often linked with the activation of microglia and astrocytes.^29^ These cell types govern the regenerative capability of severed spinal cord axons. Along with inflammation, another significant characteristic feature of SCI is glial scar formation because of astrogliosis (activation of astrocytes). These are the significant events responsible for the death of healthy oligodendrocytes and neurons around the lesion site.^30^ We have established an in vitro model of mixed glial culture prepared from the spinal cord of postnatal rats to study inflammation and changes in glycans from acute to chronic phases.

Glycans are polysaccharides present in all living organisms. They are fundamental building blocks of cellular structure, energy storage and system regulators. They form an integral part of several glycoconjugates such as glycolipid, glycoprotein, or proteoglycans.^31^ Glycans are divided into several classes: glycosaminoglycans (GAGs), N‐linked glycans, O‐linked glycans, glycosphingolipids, and glycosylphosphatidylinositol (GPI) anchor.^13^ GAGs are of several types, such as hyaluronan (hyaluronic acid, HA), chondroitin sulphate (CS), heparin sulphate (HS), keratan suphate (KS) and dermatan sulphate (DS).^14^ N‐linked glycans are processed or added into proteins/lipids in the endoplasmic reticulum to asparagine residues present proteins through an N‐acetylglucosamine (GlcNAc). There are four types of N‐glycans: high mannose, bi‐antennary, tri‐antennary and Tetra‐antennary.^32^, ^33^ O‐linked glycans are processed or added to serine, threonine, or tyrosine in the Golgi apparatus on proteins through an N‐acetylgalactosamine (GalNAc). O‐linked glycans can also be added to mannose (Man), fucose (Fuc) or glucose (Glc) residues.^34^

The O‐glycosylation and N‐glycosylation of several proteins define their functions. For example, collagens, fibrin, fibronectin, laminin, HA, etc. are either N‐glycosylated or O‐glycosylated.^35^ In CNS N‐glycans are either or both sialylated and/or fucosylated, which governs a particular function of N‐glycans. Hypersialylation is a characteristic feature in which sialyltransferase (and its other 36 enzymes) increases (polysialyltransferases). It catalyzes different sialic acid linkages such as α‐(2‐6, or, 2‐8) on glycan chains.^12^ Invertebrates, glycans usually end with a sialic acid (sialylation) on α‐3 or 6 position linkage,^13^ supporting molecular identification. Another example of O‐glycosylation and N‐glycosylation of proteins is integrins' family of receptors. These interact with the surrounding glyco‐microenvironment and help cell attachment and cell–cell communication.^36^

Glycans are ubiquitous throughout the nervous system and play crucial roles during cellular differentiation, immune cell activation and homeostasis^13^, ^37^. Therefore, it was critical to understand the role of glycans during inflammatory conditions and their expression patterns. Glial activation from the acute to the chronic phases of inflammation impacts cellular dynamics and extracellular matrix.^14^ GlcNAc moieties are essential not only in inflammation but also in glial scar formation. They are necessary for the biosynthesis of keratan sulphate, a part of glial scar.^38^, ^39^ N‐linked glycosylation and mannosylation are involved in acute and chronic inflammation.^40^

Glycans linkages expressed on glia were identified in the treatments grouped according to days (1, 7, 14, 21, and). It was observed that, in the group of cytokine combination, the signal had increased for the lectins with a higher response. This phenomenon was observed, for example, in the case of NPL, GNA, LEL, STL and DSL (day 1, 7, 14 and 21) and SNA, PSA, AAL, AAA, JAC and MOA (day 7, 14, 21), indicating higher general glycosylation in the mentioned group. The cytokine combination group would be the one that would differ from the control and LPS groups (Figure 1A–E). We observed higher expression of sialylation, fucosylation which are involved in inflammation paradigm. In addition, N‐linked acetyl glucosamine and galactosamine take part in the biosynthesis of keratan sulphate and chondroitin sulphate respectively. They are the main components of astrocytic scar formation after SCI. The higher sialyation upon cytokine treatment in the later stages of inflammation confirms the (findings of) reports where higher sialyation and fucosylation were observed in response to the inflammation.^41^ N‐acetyl galactosamine polymers are essential for synthesizing chondroitin sulphate,^42^ and therefore these glycans play a significant role during glial scar formation.

Small‐molecule inhibitors have recently received much attention in biomedical applications for biomaterial functionalization because of their relative stability and ease of distribution.^43^ STI inhibitor were tested in vitro for their ability to alter the glycome. We also noted that these compounds had no negative effects on cell viability. Sialic acid is an essential glycan for microglia and neuronal function. Sialic acid expression (hyper sialylation) acts as a crucial checkpoint for neuronal cells upon microglia‐mediated complement pathway activation, phagocytosis, oxidative stress. STI inhibits sialyltransferase, inhibiting the transfer of ‐(2, 3) and ‐(2, 6) sialic acid moieties. Therefore, MAA lectin staining showed reduced intensity in the STI treated groups. The early findings did not demonstrate a decrease in NFkB‐p65 expression, which is a key mechanism in inflammation. As a result, more studies are needed to see if STI treatment could decrease inflammation caused by cytokine combination therapy. However, as seen in cell mitro stress results, STI was able to reverse proton leak, basal respiration, spare and maximal respiration capacity altered by cytokine treatment. As these parameters are crucial in ROS production during inflammation, therfore it would be interesting further study the mechanism involve in this paradigm.

The primary function of mitochondria is to provide energy to the cell by producing adenosine tri phosphate (ATP), a cell's energy currency.^44^ Mitochondria play a role in cellular homeostasis, controlling cell growth and cell cycles, calcium homeostasis and apoptosis.^44^ Neuronal cells and glial cells rely on mitochondria's optimum functioning, and any impairment could lead to neurodegenerative disease.^45^ In addition, malfunction in the mitochondrial activities can trigger a hyper response during injury or disease conditions, which further damages healthy cells and slows the recovery.

Microglia, upon activation, depend heavily on glycolysis for their metabolism and, astrocytes depend on glycolysis and oxidative phosphorylation for their energy generation and, upon activation, they switch to glycolytic pathways for adenosine triphosphate (ATP) production.^46^ Due to activation, their metabolism increases and so does the glucose consumption. This increases lactate production via glycolysis and CO2 production via mitochondrial oxidative phosphorylation (respiration) which acidifies the medium. Therefore, both cell types have a loosely assembled mitochondrial respiratory chain which could trigger higher ROS generation and promote a maladaptive glial response.^47^, ^48^, ^49^

It has been well reported that under inflammation stimuli^50^ or several pathological conditions^51^ mitochondrial changes will have severe impacts on alleviating the disease conditions. Inflammation triggered in MGC caused mitochondrial respiration impairment as proton leak was increased and coupling efficiency was decreased. This was caused by more cells losing mitochondrial membrane potential upon cytokine combination treatment. Excessive inflammation in glia increases demands for ATP production which impairs the electron transport chain, increasing glycolysis. Due to the unbalance between energy need and capacity of mitochondria there is leakage of protons from the inner membrane of mitochondria. This was caused by more cells losing mitochondrial membrane potential upon cytokine combination treatment. Excessive inflammation in the glia increases demands for ATP production, which impairs the electron transport chain and increases glycolysis. Due to the imbalance between energy need and the capacity of mitochondria there is leakage of protons from the inner membrane of mitochondria. In the end, this leads to ROS production.^52^ This data was correlated with the proton leak parameter which determines whether there is mitochondrial membrane depolarization.^53^ It is well known that immune cells with quiescent and anti‐inflammatory phenotype primary based on fatty‐acid metabolism. In contrast, pro‐inflammatory phenotypic immune cells use glycolysis as the main source of ATP production.^54^ Coupling efficiency (an efficiency to couple proton translocation across the mitochondrial membrane to ATP production).

Sialic acid linked by ‐(2,3/6/8)‐conjugated to galactose. The NFkB pathway regulates sialic acid linkage, especially upregulating ST6Gal I mRNA over ST3GalNAc IV mRNA expression, favoring ‐(2, 6) expression over ‐(2, 3).^55^ Recent findings shown that adding sialic acid to a terminal galactose by sialyltransferase on N‐ and O‐glycans is elevated in inflammatory conditions such as (IVD degeneration).^56^, ^57^ 3Fax‐Peracetyl Neu5Ac (Sialyltransferase inhibitor) is a promising and effective molecule which blocks these sialyltransferase enzymes. Thereby it might be affecting NFkB pathway, as this pathway is crucial during neuroinflammationa and overstimulation leads to mitochondrial dysfunction.^58^ Therefore, STI treatment could effectively reduce mitochondrial dysfunction by having the effect of the NFkB pathway, which needs further investigation. As shown in Figure 6, cell mitro stress results, STI was able to reverse proton leak, basal respiration, and spare and maximal respiration capacity altered by cytokine treatment. As these parameters are crucial in ROS production during inflammation indicates that the activation of the inflammatory pathways regulates mitochondrial respiratory in a glycosylation dependent manner. The current study is the first functional investigation of glycosylation regulation in a mixed glial culture model, elucidating the role of the glycome in the progression of neuroinflammation and identifying potential therapeutic targets for future glyco therapies in neuroinflammation. Therefore it would be interesting further study the mechanism involve in this paradigm. More importantly when we treated MGC for 7 days with cytokine combination treatment (Figure 5), there was an increase in the MAA binding intensity in the cytokine combination treatment group, which was reduced upon STI treatment, which confirmed that STI could downregulate the higher expression of sialic acid during inflammation. We also acknowledge that there was no marked decrease in NFkB‐p65 expression, which is an effective element in inflammation but similar trend has been observed in other similar study which is attributed to that fact that the STI treatment has no direct effect in down regulating the signal but effects is observed on functional outputs (Figure 6).

In conclusion, Glycosylation plays a critical role during SCI progression. Therefore, in this study, glycan changes were evaluated in the mixed glial inflammatory model. First, a lectin microarray was performed which showed significant differential glycosylation from the acute to the chronic phase in a cytokine combination induced inflamed MGC model. It was found that several N‐ and O‐linked glycans associated with glia during SCI were differentially regulated. This was further confirmed with lectin immunostaining in which GalNAc, GlcNAc, mannose, fucose and sialic acid binding residues were detected in astrocytes and microglia. The LPS treated group showed lower binding than those/that of the cytokine‐combination treated group. This model can study the progression of inflammation and corresponding glycosylation changes on the glial cell surfaces. STI was able to reduce sialic acid expression in MGC and even along with cytokines. Mitochondrial function assay revealed that the impairment caused due to cytokine was significantly reversed by decreasing hypersialylation. This model can study the progression of inflammation and corresponding glycosylation changes on the glial cell surfaces.

## AUTHOR CONTRIBUTIONS

V.P. designed the study, performed all the experiments, analyzed and interpreted the data, and wrote the paper. R.B contributed to the STI and Seahorse analyzer data acquisition, and paper writing. C.W contributed to lectin stainings and paper writing. S.M. contributed to data analysis and interpretation. All authors were involved in the drafting or critical revision of the article and approved the final version. A.P. contributed to the concept, data analysis, interpretation, had full access to all the data in the study, and took responsibility for the integrity and accuracy of the conclusions.

## CONFLICT OF INTEREST

There are no financial or personal of interests.

## Supporting information


Appendix S1

## Data Availability

The data that supports the findings of this study are available in the Supplementary Material of this article. Further information and requests for resources should be directed to and will be fulfilled by the lead contact, Abhay Pandit (abhay.pandit@nuigalway.ie).
