# Supplementary material for: An insight into new glycotherapeutics in glial inflammation: Understanding the role of glycosylation in mitochondrial function and acute to the chronic phases of inflammation
Source: CNS Neurosci Ther. 2022 Nov 15;29(1):429–44. doi: 10.1111/cns.14016 (PMC9804060; doi:10.1111/cns.14016)
Supplement: Supplementary file 1 — Appendix S1 [file CNS-29-429-s001.docx]

**An insight into new glycotherapeutic in glial inflammation: Understanding the role of glycosylation in mitochondrial function and acute to the chronic phase of inflammation**

*Vaibhav Patil^1^, ^+^Raghvednra Bohara^1^, ^+^Carla Winter^1^, Michelle Kilcoyne^1,3^, Siobhan McMahon^1,2^, *Abhay Pandit^1^*

^1^SFI Research Centre for Medical Devices (CÚRAM), ^2^Anatomy, ^3^Microbiology, National University of Ireland, Galway, Ireland

^+^Equal authorship

^*^Corresponding author

**Supplementary figure 1. Comparison between Maackia amurensis Lectin MAL-I (MAA) expression in MGC by lectin microarray under control, cytokine combination and LPS treatment after 7 days of treatment.** Data are represented as mean ± SD, n= three independent experiments pulled samples run for six technical replicates, one-way ANOVA followed by multiple comparisons Tukey post hoc test, *p<0.05, **p<0.01.


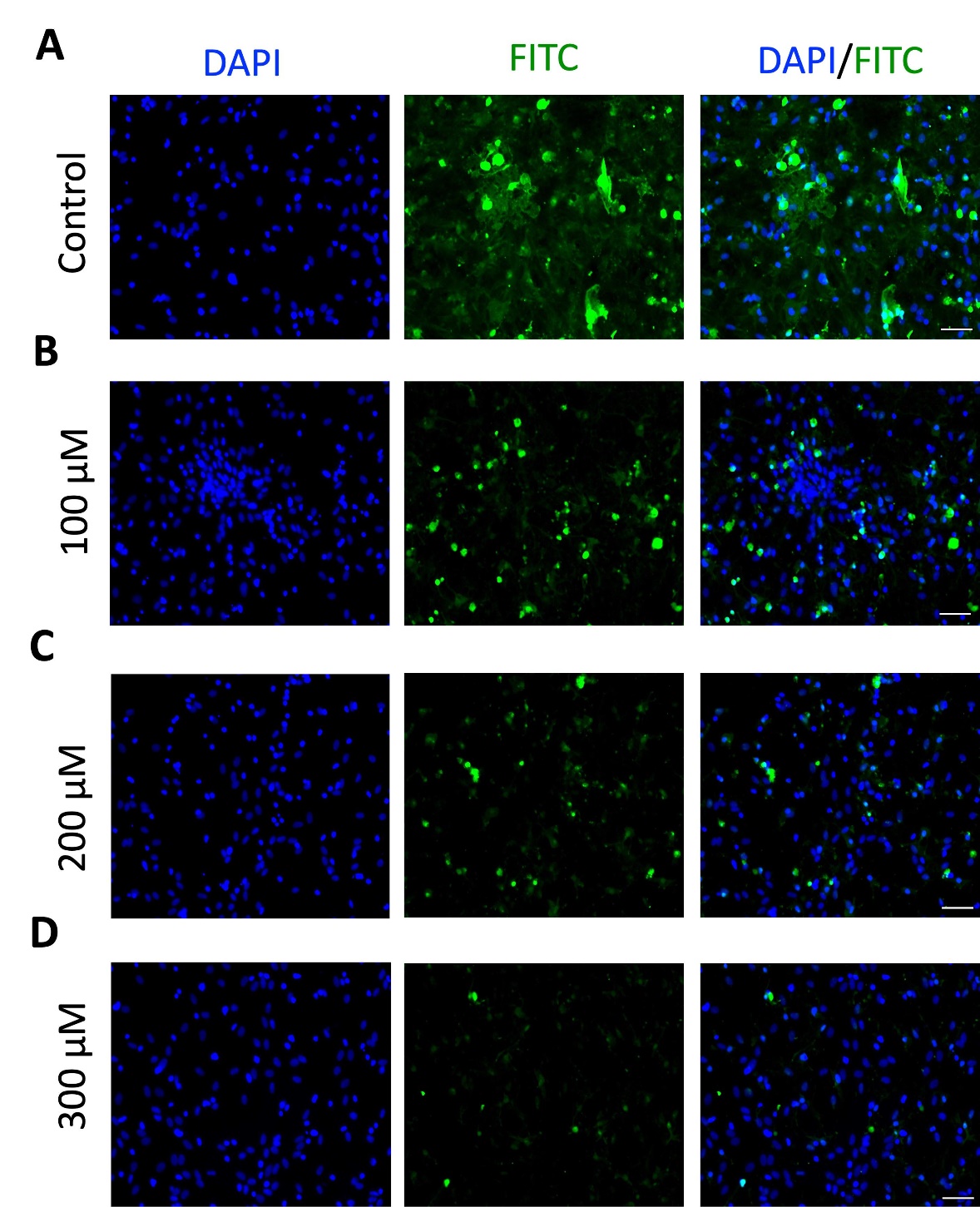


**Supplementary figure 2. Optimization of drug dose for Sialyltransferase Inhibitor, 3Fax-Peracetyl Neu5Ac (STI).** We have used three different concentrations (A) Control, (B) 100µM, (C) 200µM and (D) 300µM. Upon increase in concentration the binding affinity of MAA lectin reduces. Scale bar:50 µm.


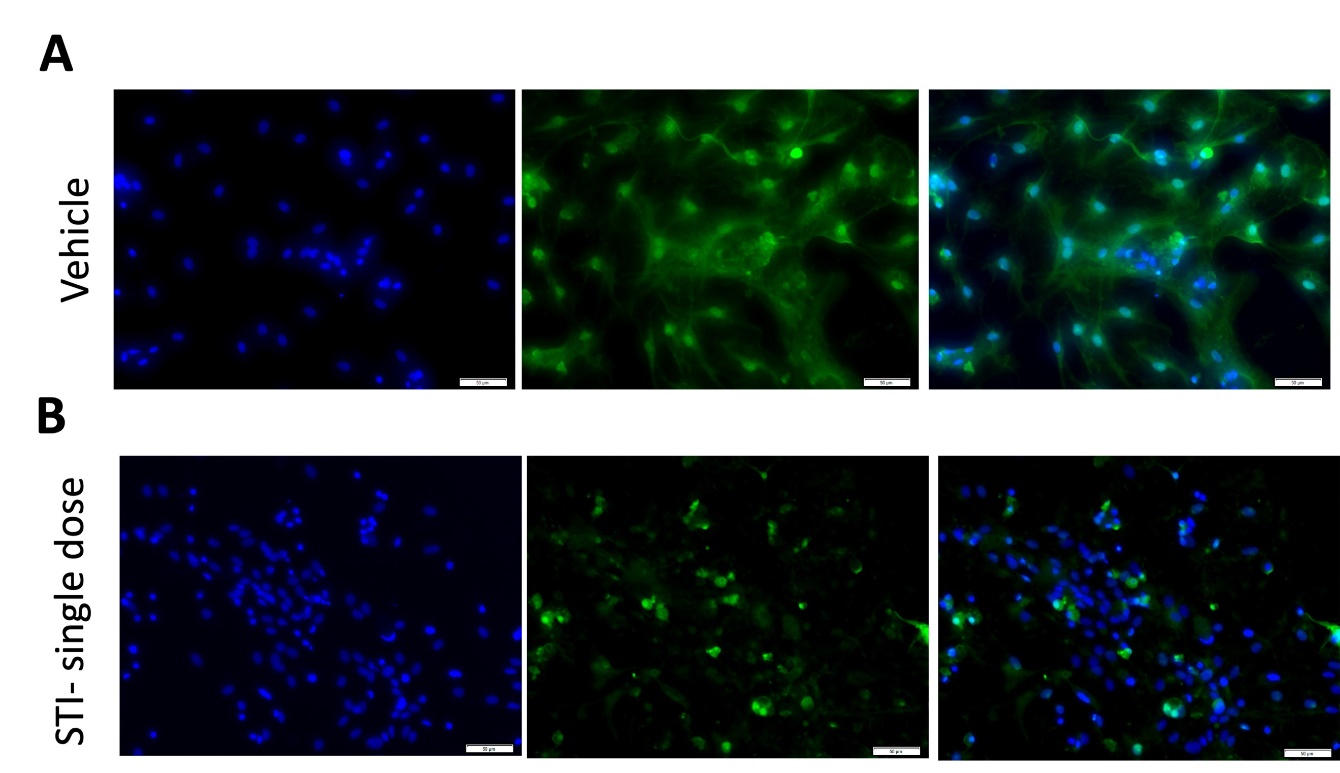


**Supplementary figure 3. Optimization of drug dose for STI.** (A) Vehicle. Experimental design: 2 x 10^5^ cells/mL of MGC were seeded in 24 well plates. After 48 hrs, the media was changed and replaced with FBS free media on day three and day six. The vehicle treatment with 300 µM concentration was given on day one and subsequently on day four. At the end on day 7 cells were stained with MAA lectin to see the expression of sialic acid. MAA recognizes α-(2,3)-linked sialic acid. and (B) After 48 hrs, cells were treated with STI at day 0 only and on day three cells were stained with MAA lectin to see the expression of α-(2,3)-linked sialic acid. Scale bar: 50 µm.


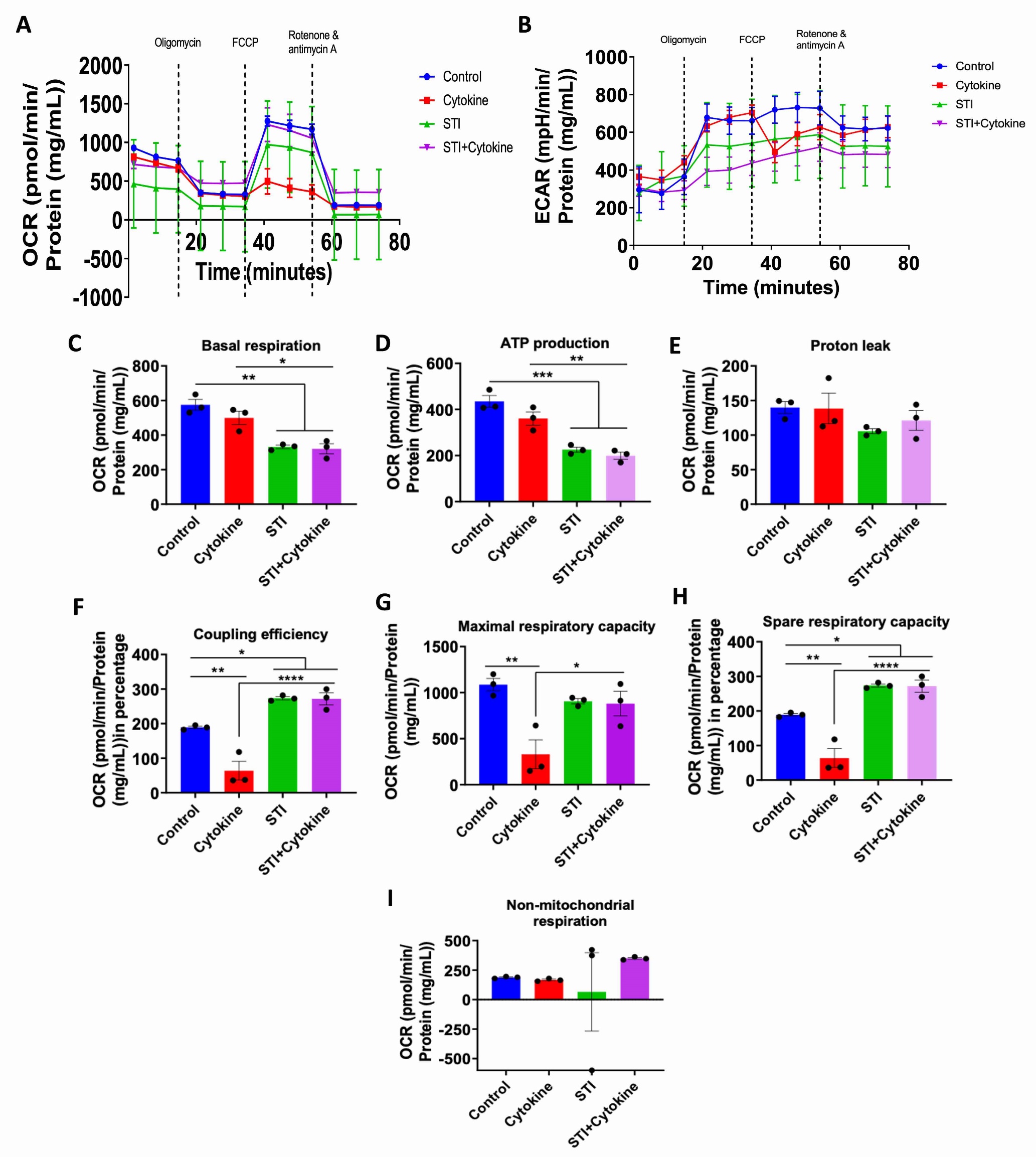


**Supplementary figure 4. A STI treatment reverts basal respiration, ATP production, proton leak, coupling efficiency, maximal respiratory capacity and spare respiratory capacity caused by cytokine treatment by day one.** After 48 hrs, MGC cells were treated with cytokine combination, with or without STI at day 0 only and on day three cells were undergone Cell Mito Stress assay. (A) OCR after the addition of three drugs (i.e. oligomycin, FCCP and Rotenone and antimycin A) sequentially. (B) Extracellular acidification rate (ECAR) after the addition of the above mentioned three drugs sequentially. (C-I) All parameters were calculated as a function of a cytokine combination and STI treatment. For this, total protein per well was calculated using a BCA protein quantification assay and data was normalised against it. Seven parameters namely, basal respiration, ATP production, proton leak, coupling efficiency, maximal respiratory capacity, spare respiratory capacity and non-mitochondrial respiration were measured and plotted as a bar graph. Data are represented as mean ± SEM, n=3. **p*<0.05, ***p*<0.01, ****p*<0.001, *****p*<0.0001. One-way ANOVA followed by multiple comparison Tukey post hoc test was performed.


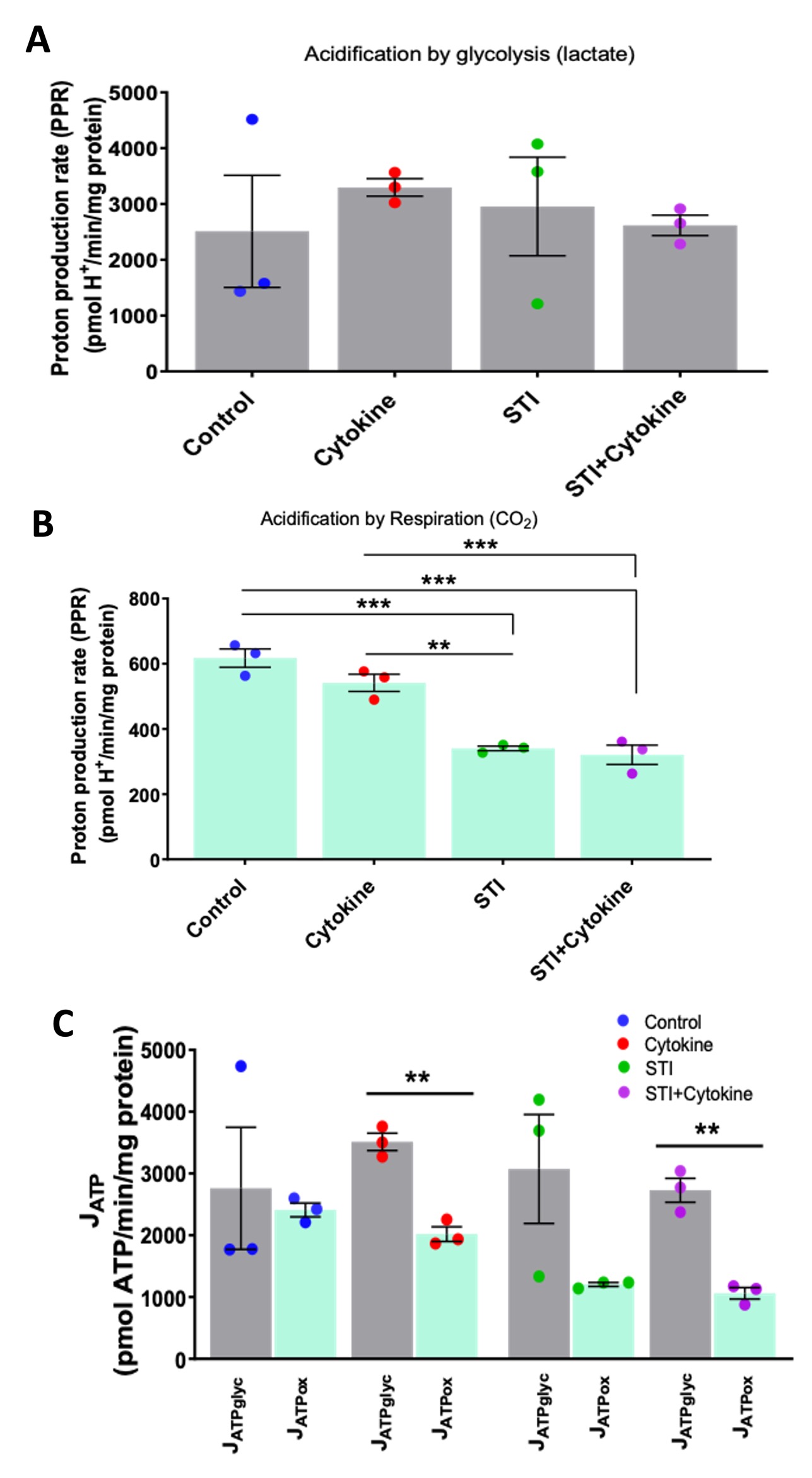


**Supplementary figure 5. Bioenergetics phenotype of mitochondrial function (at day one).** After 48 hrs, MGC cells were treated with cytokine combination, with or without STI at day 0 only and on day three cells were undergone Cell Mito Stress assay. (A) The rate of extracellular acidification caused by glycolysis due to lactate production. There was no difference in all the groups. (B) The rate of extracellular acidification due to respiration by CO_2_ production. It was higher in control and cytokine treated group compared to the STI and STI + cytokine treated groups. However, there was no difference between the STI and the STI + cytokine treatment group in acidification by respiration. (C) Data from basal ECAR and OCR has been converted to the rate of ATP production by glycolysis and oxidation using formula. The rate of ATP production by glycolysis was not significant different between groups (J_ATPglyc_). Under four treatments the rate of ATP production by glycolysis was higher than the rate of ATP production by oxidation. Data are represented as mean ± SEM, n=3. ***p*<0.01, ****p*<0.001, one-way ANOVA followed by multiple comparison Tukey *post hoc* test was performed.


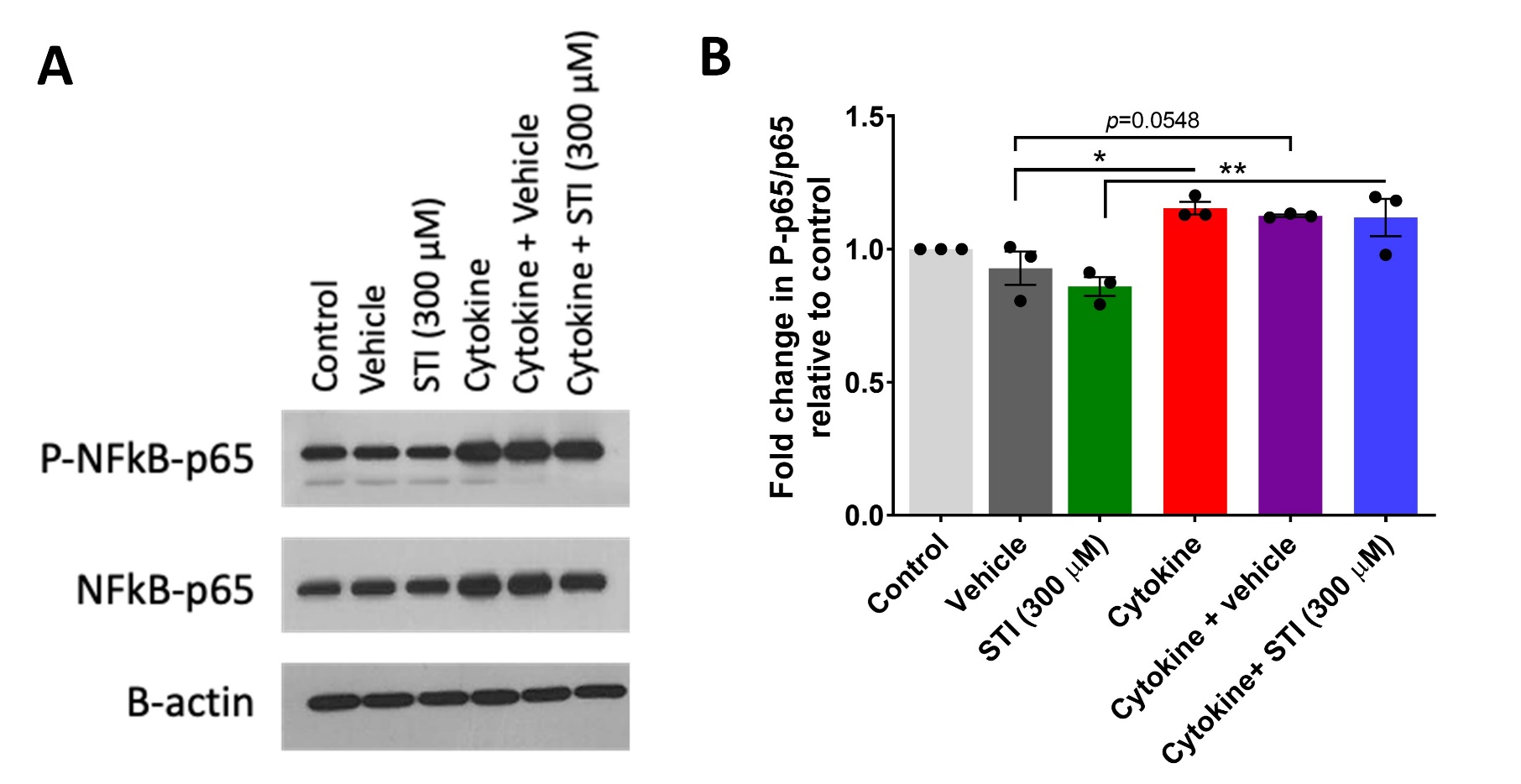


**Supplementary figure 6. Effect of STI on NFkB-p65 pathway in MGC under cytokine combination treatment.** After 48 hrs, cells were treated with STI with or without cytokine combination at day 0 only and on day three proteins were extracted and western blotting analysis was performed. (A) Western blots. (B) Quantification the intensity of the P-NFκB-p65 which was normalised to NFκB-p65 with respect to the control group. Alone STI treated group showed decreased expression of this pathway compared with all the groups with cytokine combination. Data are represented as mean ± SEM, n= three independent experiments. ^**^*p*<0.01, ^*^*p*<0.05; One-Way ANOVA, *post hoc* multiple comparison Tukey test.

**
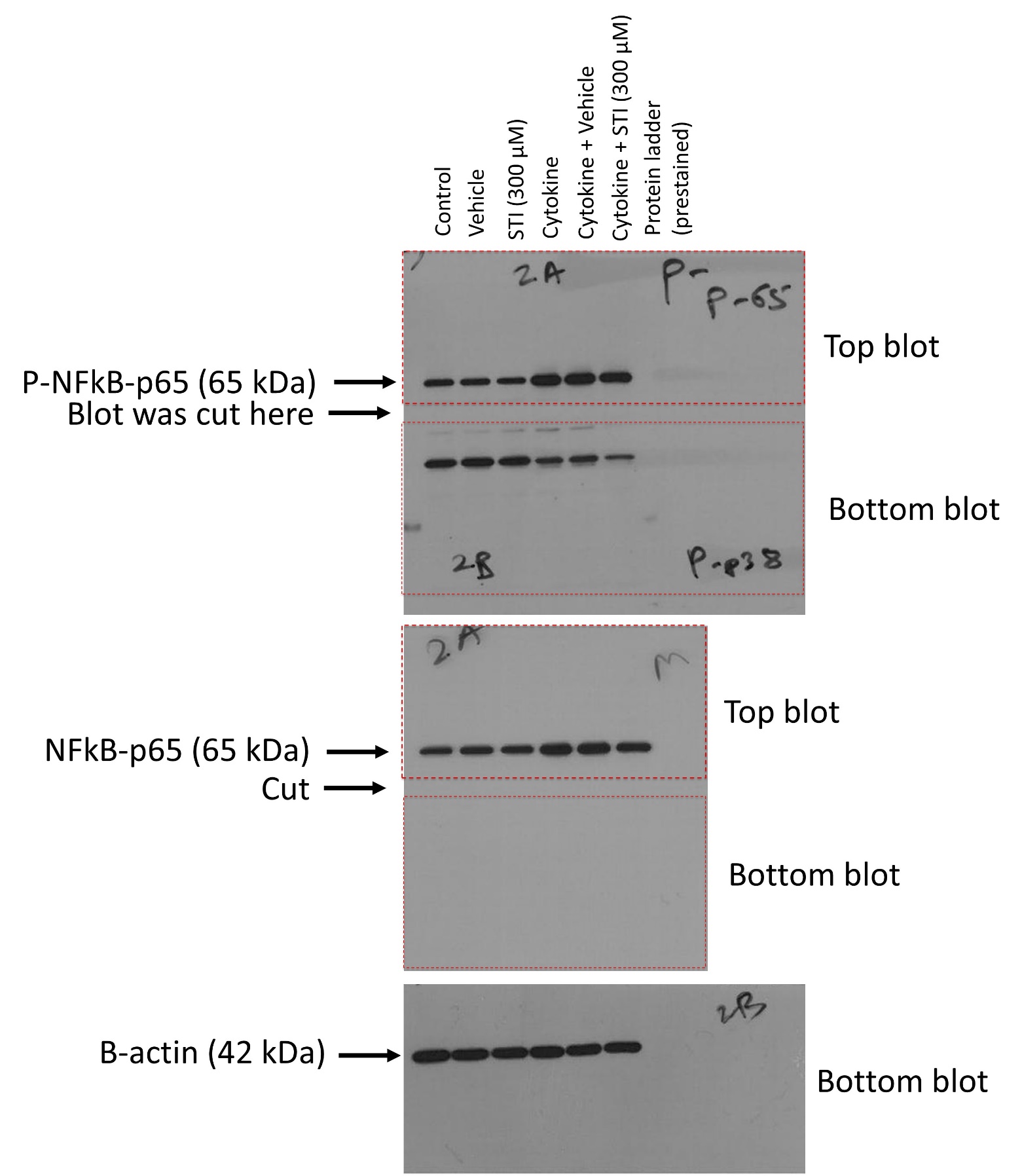
**

**Supplementary figure 7. Original blot of effect of STI on NFkB-p65 pathway in MGC under cytokine combination treatment experiment.** Antibodies against these protein are well characterized, therefore, the blot was cut preceisely to observe multiple proteins. Blot was cut at in order to assess NFkB-p65 and B-actin proteins. It shows the single band of P-NFkB-p65 protein (65kDa) and after stripping NFkB-p65 protein (65kDa) on top blot. Wheras, bottom blot was used to assess the expression of B-actin.

**Supplementary figure 8. Reduction in binding intensity after co-incubation of lectins with haptenic sugars demonstrates lectin binding is carbohydrate mediated.** All lectin staining was carried out in parallel in the presence of 100mM solution of the appropriate haptenic sugar. Competitive inhibition by these haptenic sugars resulted in a reduced binding intensity of the lectin to the glycans present in the MGC culture. The lectins and corresponding inhibitory carbohydrates were A) SNA-I in lactose, B) WFA in lactose, C) PNA in lactose, D) WGA in mannose, E) MAA in galactose, F) PHA-E in bovine IgG, and G) UEA-I in fucose. A control containing H) only TBS was conducted to determine the amount of auto-fluorescence. Finally, since the goal of this study was to do triple-staining for lectins, (GFAP and CD11b), I) secondary-antibody control was conducted to determine the amount of unspecific binding. Green = FITC, Blue = Hoechst, Red = Alexa Fluor 594, Purple = Alexa Fluor 647. Scale: 100 μm.

**
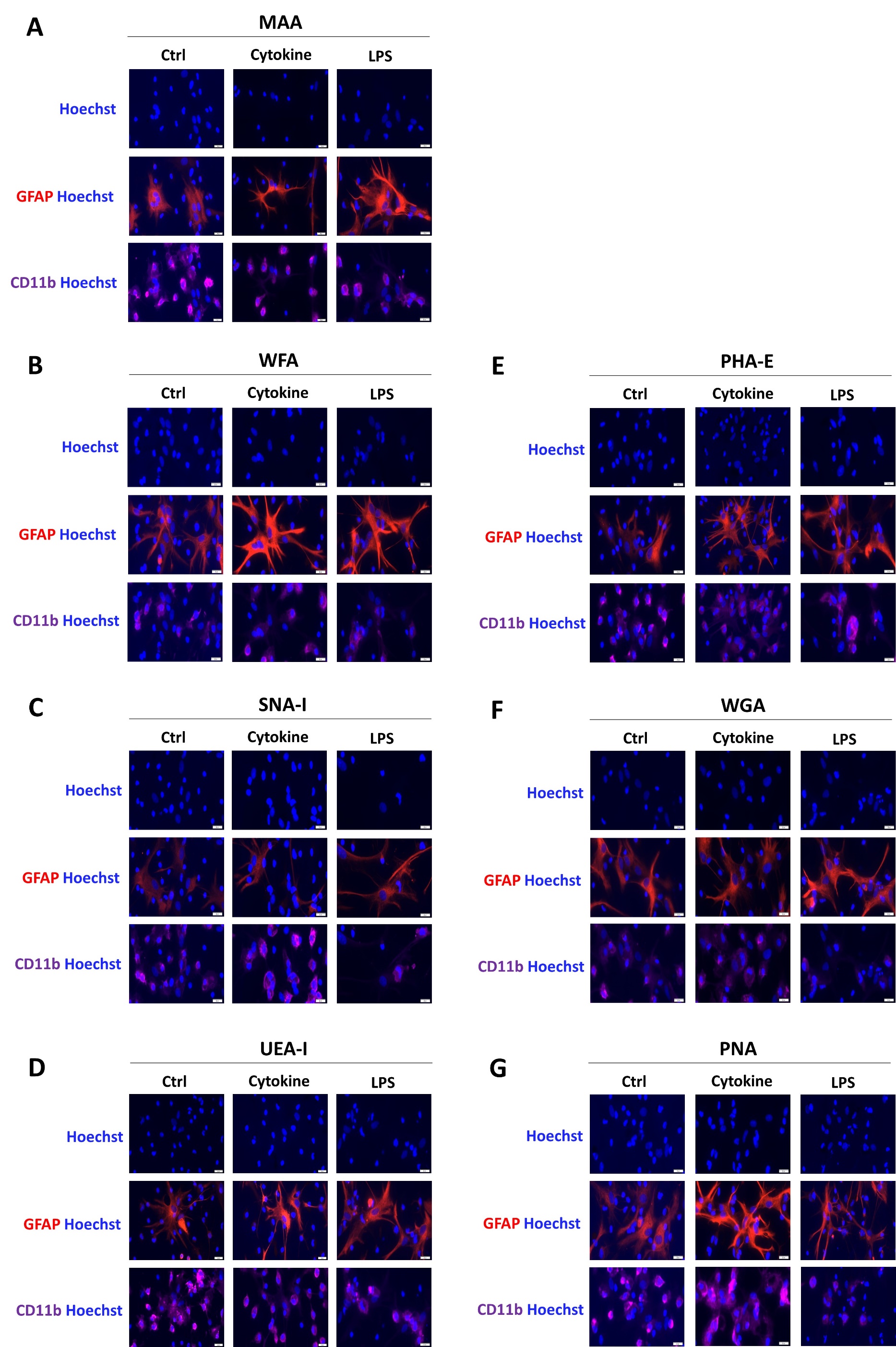
**

**Supplementary figure 9: Individual staining of astrocytes and microglia of effect of cytokine combination and LPS on the expression of lectins.** Scale bar: 20 µm.

**Supplementary Table 1.**  Lectin microarray Panel. Specificities of the lectins included in the array. Man: Mannose, GlcNAc: N-acetylgalactosamine, Gal: Galactose, LacNAc: N-acetyllactosamine, Sial: Sialic acid, Fuc: Fucose, a-Gal: α-Galactose.

**Supplementary Table 2.**  Lectin immunostaining panel.

**Supplementary Table 3.** Summary of the glycan expressions analyzed by lectin staining at day one.
